# Ultrasound description of the coelomic cavity of the axolotl (*Ambystoma mexicanum*) in a clinically healthy population: a pilot study

**DOI:** 10.1038/s41598-024-62264-z

**Published:** 2024-05-23

**Authors:** Sabrina Vieu, Ninon Le Poul, Léa Tur, Cécile Aupée, Réjane Kerbrat-Copy, Nora Bouhsina, Ophélie Cojean, Marion Fusellier

**Affiliations:** 1 Oniris, CHUV, Service des Nouveaux Animaux de Compagnie, 44300 Nantes, France; 2 Oniris, CHUV, Service Transversal d’Imagerie Médicale, 44300 Nantes, France; 3 BIOEPAR, Oniris, INRAE, 44300 Nantes, France; 4 CHU Nantes, INSERM, Regenerative Medicine and Skeleton, RMeS, UMR 1229, Nantes Université, Oniris, 44000 Nantes, France; 5 Roosevelt Veterinary Clinic, 56000 Vannes, France

**Keywords:** Herpetology, Ecology, Evolution

## Abstract

Axolotls *(Ambystoma mexicanum)* are extensively studied for their relevance in human medical research. Despite being critically endangered in the wild, they have gained popularity as household pets. Although they have been kept in captivity for over a century, detailed descriptions of their coelomic organ anatomy remain limited. Also, this species exhibits significant variations compared to other amphibians. Ultrasound is a non-invasive and painless medical imaging technique, ideally suited for investigating internal organs or structures. This study focused on describing the ultrasound appearance of the axolotl coelomic cavity. It details the identification, localization and parenchymal description of major organs in 28 neotenic axolotls using ultrasound frequencies ranging from 7 to 15 MHz. The accuracy of the results was validated by comparing ultrasound findings with necropsy results from one male and one female axolotl. The heart, lung surface, liver and reproductive tracts were visualized. Measurements, along with confidence intervals, were calculated for the spleen, kidneys, testicles, gastric wall, gallbladder, and pylorus. Occasional detection of hyperechoic millimetric particles in the gallbladder or ascites was noted. However, visualization of the pancreas and bladder was not possible. This research outcomes involve the development of a comprehensive atlas comprising images obtained throughout the study. Additionally, the experiment established a reproducible and readily accessible protocol for conducting anatomy-morphological assessments in axolotl medicine. This protocol stands as a crucial preliminary stage before advancing to lesion identification.

## Introduction

Amphibians represent the most imperiled vertebrate class due to a myriad of factor, including habitat loss and degradation, the impacts of climate change, and disease.^1^ The axolotl (*Ambystoma mexicanum*) which belongs to the order Caudata (or Urodela) and the family Ambystomatidae, is a critically endangered species endemic to its natural habitat in Mexico’s Xochimilco Valley.^2–5^ Global warming and poor water quality, stemming from regional aquaculture and human pollution, stand as primary contributors to habitat loss and fragmentation.^6–8^ The wild population of axolotls is estimated to range from 50 to 1,000 individuals.^4^ To address the conservation needs of amphibians and ensure the survival of species into the future, breeding programs are often deemed necessary.^9^ Consequently, captive breeding and release initiatives have been established for the endangered urodele species, including *Ambystoma mexicanum*.^10^.

The axolotl has also emerged as a primary focus of research since the twentieth century, garnering considerable interest in embryonic development mechanisms, aging processes, and organ regeneration.^2,3,6,11,12^ Research has particularly delved into its metamorphic capacity, with studies examining thyroxine synthesis by the thyroid gland.^3,13,14^ Additionally, the axolotl exhibits numerous unique characteristics, including the ability to undergo neoteny or pedomorphosis. This phenomenon involves incomplete metamorphosis, wherein animals retain larval characteristics even after reaching sexual maturity.^15^ This trait enables individuals to reproduce and be maintained in a larval stage within aquariums, contributing to their popularity in both laboratories and among owners.^16^ As such, axolotls have secured a place as pets and attractions in zoological park, and their utilization in medical research is progressively expanding worldwide.^17,18^ Although axolotl breeding is very common, captive specimens mainly originate from 34 individuals imported into Europe.^3^

Ultrasonography offers a non-invasive, safe, and user-friendly method, making it the preferred choice in research, as well as in human and veterinary medicine.^19–21^ Performing a thorough ultrasonographic examination of the coelomic cavity necessitates a comprehensive understanding of amphibian anatomy, with general descriptions of axolotl internal organs dating back to the nineteenth century.^22–24^ Indeed, amphibians exhibit various distinctive features, such as the absence of a diaphragm, resulting in a singular cavity known as the coelom, instead of separate thoracic and abdominal regions. This implies that the heart is in close proximity to the liver. Additionally, the major coelomic organs of the axolotl include the heart, lungs, spleen, liver with gallbladder, digestive system comprising the stomach and intestine, kidneys and gonads.^25,26^ By taking ethical considerations into account, ultrasound facilitates the exploration of organs and monitoring of diseases in amphibians without causing pain.^25,27–29^ This capability proves valuable for both wild populations and pet axolotls, enabling the potential detection and monitoring of diseases and facilitating the initiation of treatment. Furthermore, ultrasonography can aid in the examination of reproductive organs and processes, thereby supporting conservation efforts for wild populations and breeding programs for pet amphibians.^30^.

Hence, our study endeavors to establish ultrasonographic standard measurements of axolotl internal organs by: (i) offering precise descriptions of the position, ratios, and size of healthy organs within the coelomic cavity; (ii) developing resources encompassing general anatomy and experimental guidelines tailored specifically to the axolotl, which will prove invaluable to scientists conducting research in this area.

## Materials and methods

### Study population and data collection

Students, employees and client-owned axolotls were solicited from a veterinary teaching hospital between September 2022 and December 2022.

Axolotls underwent an initial assessment to ensure their overall health. Inclusion criteria comprised an age of 1.5 years, the absence of clinical signs, and adherence to appropriate diet and environmental conditions as per general recommendations for axolotls.^31^ The animals underwent a 3-day fasting period prior to diagnostic imaging to clear their gastrointestinal systems. Owners were instructed to transport the animals in a water tank from the aquarium with a temperature range of 15.6–17.8 °C (60–64 °F).

The animals were acclimated for 1 day before undergoing ultrasound examination and were housed at the Exotic Pet Department of the veterinary teaching hospital for the duration of the protocol. Upon arrival, each axolotls’ tank was labeled with a unique identification number. Throughout the study, the animals were housed individually in 10L plastic containers with water from their respective aquariums, maintaining a 12-h light/12-h dark cycle.

**Figure 1:**
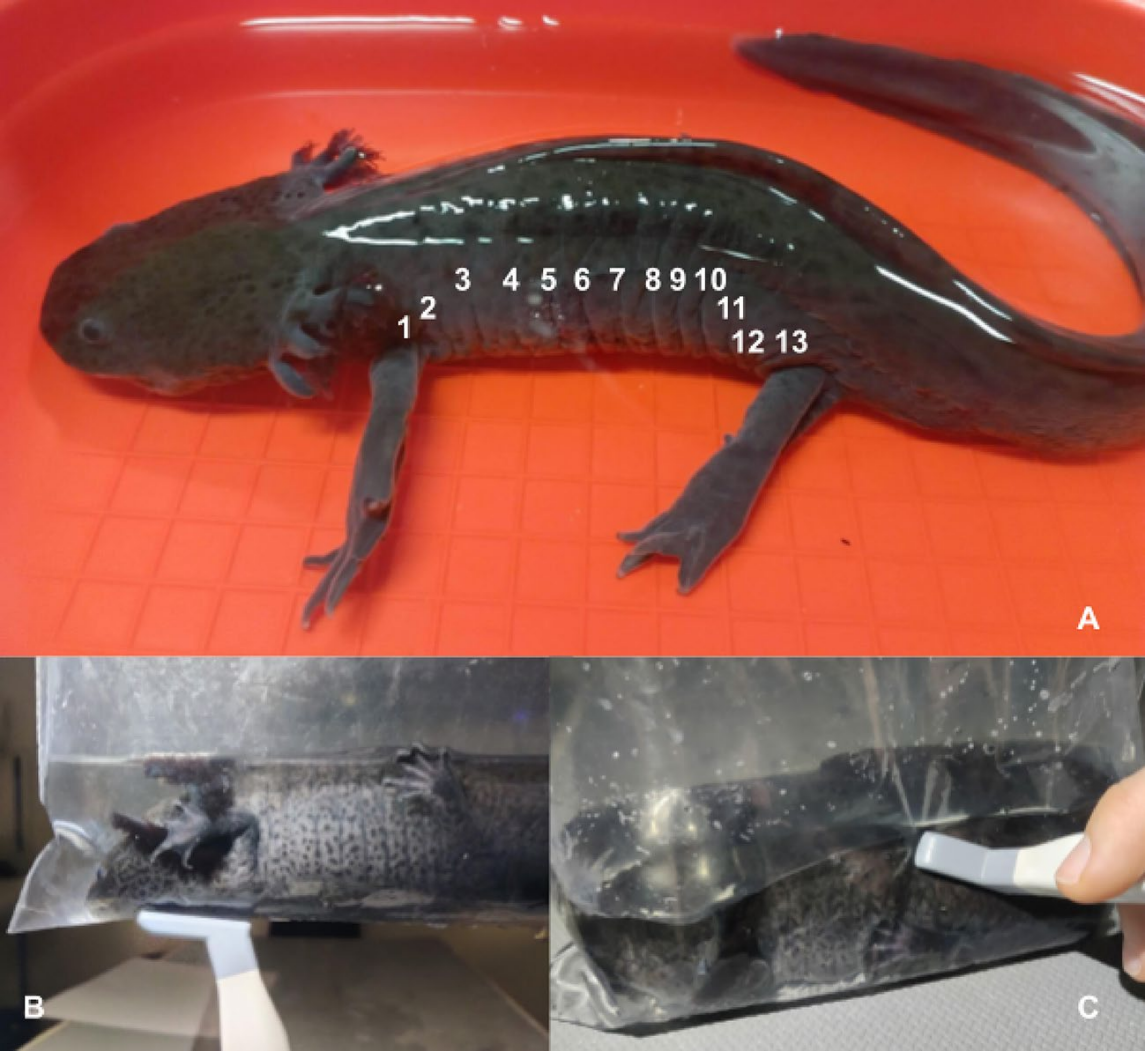
(**A)** Distant clinical examination and counting of costal grooves in an axolotl. (**B)** Ventral placement of the transducer for ultrasonographic examination in an axolotl placed in a plastic bag filled with water from its aquarium. (**C)** Dorsolateral placement of the transducer for ultrasonographic examination in an axolotl placed in a plastic bag with water from its aquarium.

Experimental manipulation of the axolotls commenced on the following day, with great care. All procedures were conducted using rinsed disposable latex gloves. During the clinical examination, the axolotls were weighed, their skin and gills were examined, body length was measured, and the number of costal grooves was counted (Fig. 1A). Additionally, their displacement and buoyancy in water were observed. Owners were contacted at regular intervals (1 week, 3 months, and 6 months after ultrasonography) to confirm the animal’s health. The same clinical examination performed before ultrasonography at veterinary teaching hospital was repeated during these follow-up evaluations.

### Protocol

The ultrasound examinations were conducted at the Diagnostic Imaging Unit of the veterinary teaching hospital by three trained operators. The examinations were conducted in a quiet room with subdued lighting, and the operators wore rinsed latex gloves throughout the procedure. All coelomic ultrasonography procedures were performed using a compact linear ultrasound transducer (L15-7io; Koninklijke Philips NV, Amsterdam, Netherlands) with a frequency range of 7 to 15 MHz. The transducer measured 3 cm in length by 1 cm in width.

For ultrasonography in aquatic species, the surrounding water serves as a medium for propagating ultrasound waves between the probe and the object being examined. To facilitate the examination, it was performed without direct skin contact and without the need for sedation or physical restraint. Axolotls were gently transferred to single-use 2.5-L smooth, transparent plastic bags filled to a quarter of their height with water from their own containers. During the ultrasound examination, ultrasound gel was applied directly onto the plastic bag. Ventral approaches were achieved by lifting the bag, while dorsal approaches were achieved by placing the bag on the examination table and maintaining upward tension. This ensured that the axolotls remained fully immersed in water throughout the examination process (Fig. 1B, C). Whenever an acoustic window of an organ was available, the costal grooves were counted. To optimize visualization of the different organs, the ultrasound probe was rotated and placed on both short and long axes as deemed convenient by the operator. Close attention was paid to any signs of stress or discomfort exhibited by the axolotls. Signs of animal discomfort included movement in water or loss of tonus (absence of movement). The duration of each ultrasonographic examination was noted for every individual animal. Additionally, ultrasound facilitated the measurement of axolotl heart rates. To minimize the duration of this manipulation and reduce the overall examination time, the number of heart beats occurring over a 15-s period was counted, and this value was then multiplied by four to obtain the number of beats per minute (bpm). A standardized protocol was established to specify the sequence and approach (ventral, lateral or dorsal) of each organ. Standardization minimized errors and guaranteed comprehensive coverage of all relevant structures. The detailed protocol is outlined in the final ultrasound atlas (Supplement 2). Following the examination, axolotls were promptly returned to their individual plastic containers.

### Data recording

As per the preestablished ultrasonographic protocol, sagittal and transverse images of each coelomic organ were captured, along with cine loops to document the dynamic aspects of the coelomic structures. All datasets were stored using available software (PACS Synapse V5; FujiFilm Corp), with each axolotl tank number serving as a unique identifier. This approach facilitated retrospective analysis of the images and served to streamline the ultrasonography process. Key parameters such as visibility, size, localization, and echogenicity were recorded for each organ. Measurements were performed by consensus among all operators using electronic calipers within the same medical image analysis software. Length was defined as the distance between the most cranial and the most caudal parts of an organ; height was measured from the most dorsal to the most ventral aspects. Width was assessed as the distance between the right lateral and left lateral margins of the body. For kidneys and testes, measurements were taken in both sagittal and transverse sections. In sagittal views, the length and height were measured, while in transverse sections, the width and height were assessed. The height of the spleen was measured in longitudinal views. Regarding the stomach, pylorus and gallbladder, wall thickness was measured perpendicular to the wall and recorded accordingly.

### Statistical analysis

The data are presented as mean, standard deviation, minimal, and maximal values for each organ measurement. All parameters were found to follow a normal distribution, and Student’s *t*-test was used to calculate the 95% confidence interval of the mean for each set of values obtained. Descriptive statistics were computed for each coelomic organ measurement using Microsoft Excel software (Microsoft Corporation, Redmond, Washington, USA).

### Ethical statement

This study received specifical approval from the Oniris Veterinary Clinical and Epidemiological Ethics Committee (no. CERVO-2022-8) and was conducted in strict adherence to the Guiding Principles for the Care and Use of Research Animals, which includes following the ARRIVE guidelines.^32^.

### Postmortem examination

During the experiment, two axolotls died while in the care of their owners. These two axolotls were housed separately from the other axolotls involved in the experiment. One 5-year-old male exhibited signs of mycosis on its gills, while one 8-year-old female was observed to have lens opacity. Due to their melanistic nature, specific dermatologic signs such as petechiae could not be observed. A postmortem examination was conducted, revealing nodules on the spleen of the axolotl with mycosis. The spleen from this axolotl was removed and discarded from the healthy organs.

**Figure 2:**
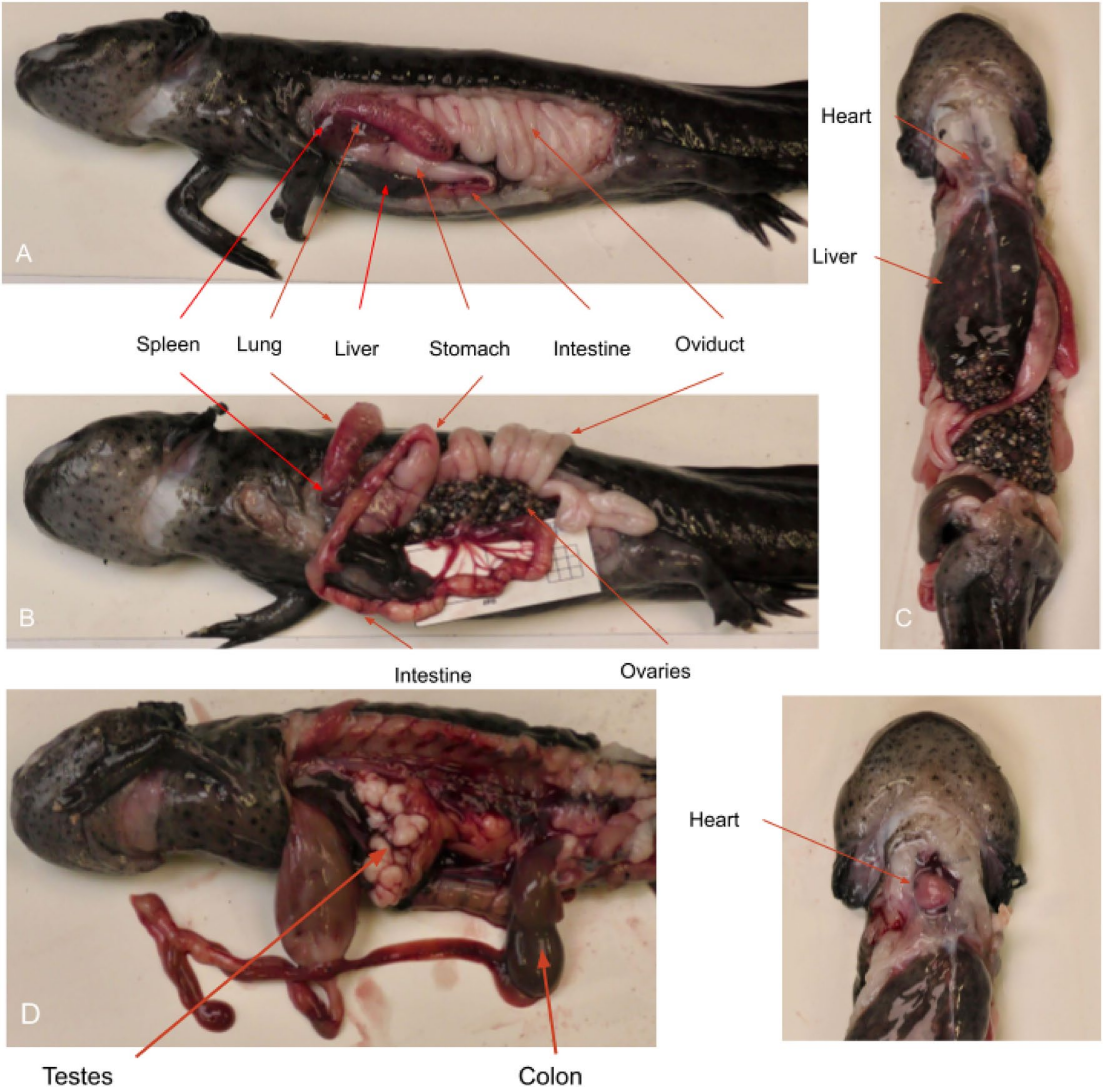
Axolotl necropsies (**A)** Photograph illustrating the appearance of coelomic organs in a female axolotl at necropsy with a left lateral approach. (**B)** Photograph illustrating the appearance of coelomic organs in a female axolotl at necropsy with a left lateral approach, with the left oviduct and lung removed. (**C)** Photograph illustrating the appearance of coelomic organs in a female axolotl at necropsy with a ventral approach. (**D)** Photograph illustrating the appearance of coelomic organs in a male axolotl at necropsy with a left lateral approach, with the left oviduct and lung removed. (**E)** Photograph illustrating the appearance of coelomic organs in an axolotl at necropsy, with highlighting of the heart.

Photographs of healthy macroscopic coelomic structures were obtained and compared with ultrasonographic findings. Additionally, ultrasonography was performed on the large internal organs to verify topography and sonographic findings, including the heart, liver, gallbladder, spleen, gastrointestinal tract, lungs, kidneys, and gonads. Scaled photographs of the large organs captured (Fig. 2). Following the ultrasonographic examination, organs were carefully dissected and inspected for any abnormalities. No macroscopic abnormalities were observed in the parenchyma of the examined organs. Thyroid glands, fat bodies, bladder, pancreas, and adrenals glands were not macroscopically detected and were therefore excluded from our results.

### Atlas creation

Schematic drawings depicting the topographic anatomy of the coelomic organs were created based on necropsy findings. For this purpose, a Wacom Intuos S device was utilized in conjunction with Adobe Photoshop Elements 2023 software.

## Results

Twenty-nine live individuals underwent initial clinical examinations, sourced from three distinct owners. A total of 28 axolotls, comprising 13 males and 15 females with different color variants (including wild type, leucistic, and albino), met the final inclusion criteria for the study, one specimen being excluded due to being too young. Two deceased axolotls were not considered in the subsequent results. The owners were contacted up to 6 months after the experiment regarding the general health of the axolotls, and they reported no abnormalities. Morphometric results are as follows: body mass = 69.19 ± 31.06 g (gr), total length = 21.93 ± 3.32 cm (cm), heart rate = 45.3 ± 11.7 beats per minute (BPM) and 13.3 ± 1.03 costal grooves (Table 1). Each animal underwent an ultrasonographic examination lasting 15 to 20 min, during which no obvious signs of stress were observed. There were no adverse effects noted during or after coelomic ultrasound examinations. Macroscopically identified coelomic organs observed during necropsies included the heart, surface of the lungs (visceral pleura), liver, gallbladder, spleen, stomach, pylorus, kidneys, testes or ovaries, and the oviducts in the female genital tract, all of which were visualized in every animal. The presence of gas within the lungs or gastrointestinal tract content hindered the visualization of the entirety of certain organs, such as the cranial part of the kidneys or testes. Counting of costal grooves counting was only feasible for lighter individuals due to the brightness of the room.

**Table 1:** Mean, median, standard deviation, minimum and maximum values of weigh, length, costal grooves, and heart rate in sampled axolotls (*n* = 28).

Individual	Sex	Length (cm)	Weight (gr)	Number of costal grooves	Cardiac rate (bpm)
1	M	X	X	X	X
2	M	30.0	140	13.0	X
3	F	28.5	190	15.0	X
4	M	18.0	42	13	52
5	M	18.0	43	12	48
6	F	17.5	51	15	28
7	F	17.5	39	14	28
8	F	17.5	43	13	52
9	F	18.5	50	14	32
10	F	19.0	59	14	24
11	M	20.0	63	13	26
12	F	20.0	81	13	26
13	F	24.0	62	13	40
14	M	22.0	72	13	40
15	F	23.0	57	11	52
16	F	24.0	60	13	48
17	F	23.0	83	12	60
18	M	24.0	78	15	NR
19	M	22.5	56	13	60
20	F	23.5	74	15	52
21	M	24.0	71	13	56
22	M	23.0	65	14	56
23	F	20.0	53	14	52
24	M	24.5	71	13	56
25	F	26.0	82	14	48
26	M	23.0	63	12	48
27	M	22.0	65	12	52
28	F	19.0	55	13	52
Minimum value		17.5	39	11	24
Maximum value		30.0	190	15	60
Mean		21.93	69.19	13.30	45.33
Standard deviation		3.32	31.06	1.03	11.70
Median		22.3	62.5	13.0	48.0

Schematic drawings depicting the topographic anatomy of the coelomic organs have been created and are accessible in Supplement 1. Additionally, an ultrasonographic atlas of the axolotl coelomic cavity is provided in Supplement 2.

### Ultrasonographic anatomy

#### Heart

The heart was consistently visualized in all axolotls, typically located in the cranial part of the coelomic cavity cranially or anterior to the thoracic limbs. Within the pericardium, a small amount of fluid was physiologically present, appearing hypoechoic in comparison to different parts of the heart. A previous study has detailed echocardiographic slices and probe positioning in axolotls, enabling observation of the different heart chambers during cardiac phases. In line with the probe placement described in that article, we performed examinations using four distinct views.^24^ The heart parenchyma exhibited a uniform appearance with slightly hyperechoic walls throughout all cardiac cavities. Blood vessels were identifiable as tubular structures with thin hyperechoic walls, while the blood itself appeared hypoechoic with echogenic mobile particles.

The midline long axis was obtained through a longitudinal ventral approach at the base of the neck. This positioning allowed partial visibility of both atria on the acoustic window. Subsequently, the ventricle long axis was obtained by slightly moving the probe to the right, providing a view of the ventricle and the emerging aortic trunk. Blood flow from the ventricle to the aortic trunk manifested as a hypoechoic appearance accompanied by echogenic mobile particles. The atria long axis was observed by shifting the prove leftward from the ventricle long axis view, facilitating the visualization of atrial and ventricular diastole, as well as caudal vena cava. A 90° rotation was necessary to obtain the ventricle short axis view, which allowed for the observation of systole and diastole within the ventricle. In this final view, the sinus was easily and consistently observable.

During the ultrasonography procedure, axolotls occasionally exhibited sight movement, posing a challenge to the precise examination of the heart.

#### Liver and gallbladder

**Figure 3:**
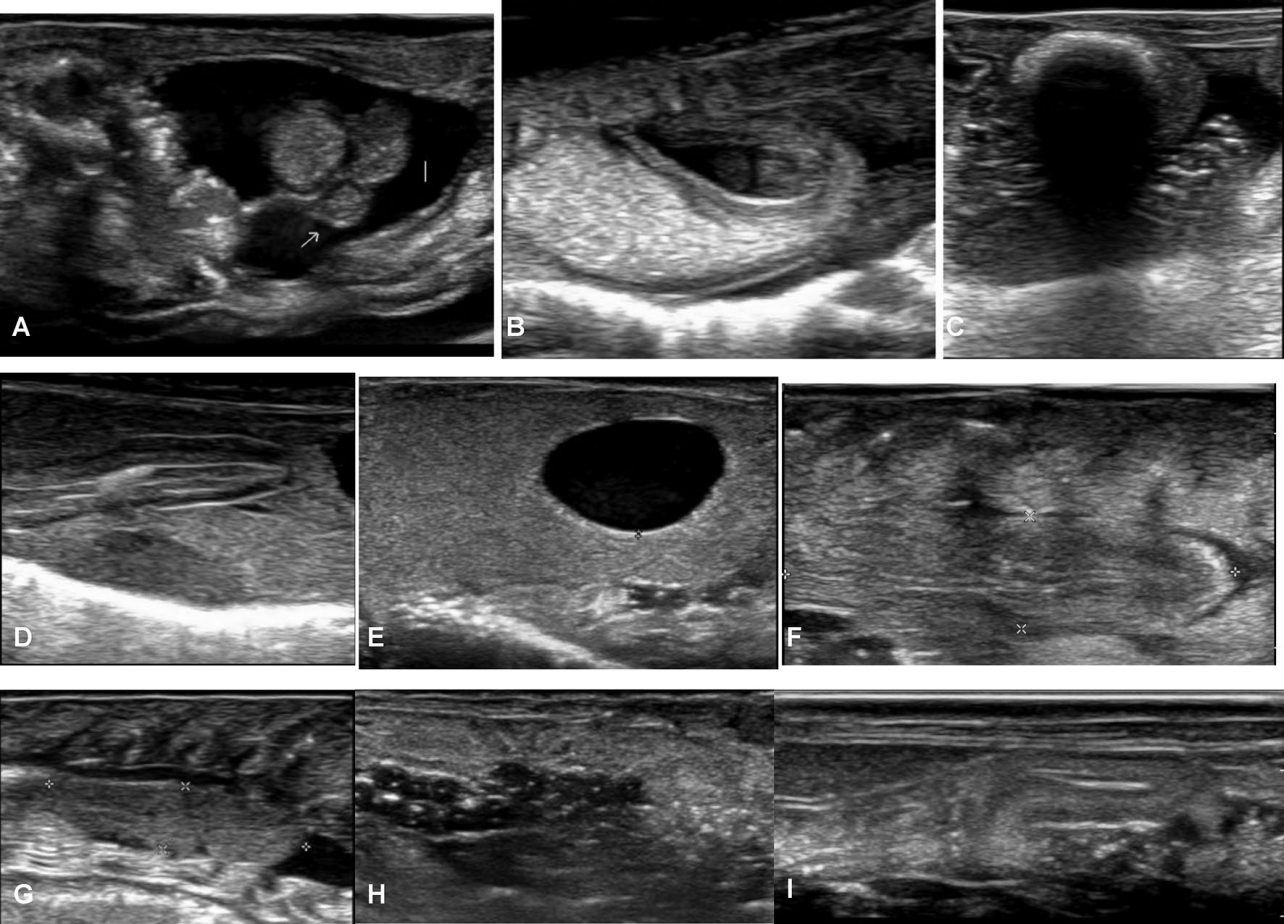
(**A**) Stomach with gastric contents in transverse axis (**B**): Stomach and intestine in longitudinal view, (**C**): Colon in transverse view, (**D**): Spleen in longitudinal view, (**E**): Liver and gallbladder in longitudinal views, (**F**) Right kidney in longitudinal view (**G**) Right testis in longitudinal view, (**H)** Right ovary in longitudinal view, (**I**) Right oviducts loop in longitudinal view.

The liver and gallbladder were consistently visualized in all axolotls. Positioned caudally to the heart, cranially to the intestine, ventromedially to the stomach, and ventrally to the lungs, the liver required a ventral approach for observation. In 12 out of the 13 axolotls where organs could be positioned with costal grooves, the liver did not extend caudally beyond the 9th groove (Table 2). Due to the organ protruding out of the ultrasound field, precise measurement was not feasible. Both long and short axis views were conducted. The liver parenchyma appeared homogeneous and ranged from isoechoic to hyperechoic in comparison to the spleen (Fig. 3D). The caudal vena cava, coursing towards the heart, could be identified as a tubular structure with hyperechoic walls and with the blood appearing hypoechoic with echogenic mobile particles within the liver parenchyma.

**Table 2:** Costal grooves counting with the mean and standard deviation of the cranial (Cra) and caudal (Cdl) positions of organs.

Individual	Liver		Spleen		Gallbladder		Stomach		Pylorus
	Cra	Cdl	Cra	Cdl	Cra	Cdl	Cra	Cdl	
4	1	7							
7	1	8					1	8	
8	1	11	1	7	8	9	2	10	
9	1	7	2	4	6	6			7
10	1	7			5	5	4	5	
11	1	6					6	10	
12	1	6	3	5	5	6			
13	1	6	3	6	4	5			
14	1	9	2	7					7
15	1	6	3	6					
16	1	8	3	4			4	5	
17	1	8			6	7			
18	1	8	3	7	6	7			
Mean	1.0	7.5	2.5	5.8	5.7	6.4	3.4	7.6	7.0
Standard deviation	0.0	1.5	0.8	1.3	1.3	1.4	1.9	2.5	0.0

The gallbladder was visualized in all axolotls using a right paramedian ventral approach. In 12 out of the 13 axolotls where organs could be positioned with costal grooves, it was consistently located between the 5th and 6th groove (Table 2). Structurally, the gallbladder appeared as an anechoic, round-shaped structure, delineated by a thin, regular echoic wall (Fig. 3E). The thickness of the wall was measured in 22 out of 28 axolotls, as the gallbladder was collapsed in the remaining specimens. Mean thicknesses are provided in Table 3**.** Additionally, hyperechoic particles were observed within the gallbladder in 9 axolotls.

**Table 3:** Mean, median, standard deviation, minimum, and maximum values (in mm) with 95% confidence intervals of the coelomic ultrasound measurements in 28 axolotls.

		Minimum value	Maximum value	Mean	Confidence interval 95%	Standard deviation	Median	Number of individuals included
Spleen height		1.5	5.9	3.3	]2.7; 3.9[	1.21	3	19
Stomach wall thickness		0.4	1.4	0.81	]0.69; 0.94[	0.27	0.8	20
Gallbladder wall thickness		0.2	0.7	0.36	]0.30; 0.42[	0.13	0.3	22
Pylorus diameter		1.0	3.0	1.76	]1.46; 2.05[	0.55	1.6	16
Right kidney	Long axis: length	9.4	22.2	15.0	]13.7; 16.3[	3.1	15.0	25
	Short axis: width	3.5	10.2	6.1	]5.5; 6.7[	1.5	6.1	27
	Height	2.7	9.2	4.9	]4.2; 5.5[	1.6	4.9	27
Left kidney	Long axis: length	10.0	21.3	15.3	]14.0; 16.6[	3.3	14.9	27
	Short axis: width	3.6	9.9	6.1	]5.4; 6.7[	1.5	5.4	25
	Height	3.0	8.6	5.7	]5.1; 6.4[	1.6	6.0	28
Right testis	Long axis: length	7.3	17.8	12.5	]8.62; 16.35[	4.18	11.9	7
	Short axis: width	3.7	7.0	5.1	]3.89; 6.36[	1.33	4.9	7
	Height	2.2	4.6	3.6	]2.83; 4.27[	0.78	3.9	7
Left testis	Long axis: length	7.0	18.6	12.6	]8.94; 16.18[	4.33	12.4	8
	Short axis: width	3.5	6.6	5.3	]3.94; 6.56[	1.25	5.5	6
	Height	3.3	5.6	4.3	]3.77; 4.89[	0.67	4.3	8

#### Spleen

The spleen was observed in all axolotls and was consistently located in the left cranial coelom, positioned laterally to the liver. It was predominantly observed between the 2nd and 7th costal grooves in 8 axolotls (Table 2), ventrally adjacent to the left lung. The splenic parenchyma exhibited a homogeneous, slightly granulated appearance and ranged from isoechoic to hypoechoic in comparison to the hepatic parenchyma (Fig. 3D). Confidence intervals for the splenic height were measured in 19 axolotls and are detailed in Table 3.

#### Stomach and pylorus

The stomach was observed in all axolotls and was consistently located to the left to the liver in the cranial and middle coelom regions. It made contact with the spleen along almost its entire length. The probe positioning for stomach ultrasound was similar to that used for liver examination. The gastric wall exhibited a layered structure, which could be visualized as follows: a hyperechoic layer, likely corresponding to the interface between the stomach lumen and the mucosa; a hypoechoic to anechoic band, corresponding to the mucosa; a hyperechoic layer, presumed to correspond to the submucosa; and a final nearly anechoic layer, corresponding to the muscularis (Fig. 3A). The thickness of the stomach wall was measured on 20 axolotls, with values ranging from 0.4 mm to 1.4 mm. In our sample, the average gastric thickness measured on a full stomach was 0.81 mm (Table 3). Gastric replenishment varies in shape and size depending on its content. Despite the axolotls being in a fasting state, some exhibited a full stomach at the time of examination. This can be attributed to the fact that axolotls ingest their excrements and eggs. Ingested eggs were identified and characterized by a hyperechoic spherical structure surrounding an anechoic core (Supplement 2).

The stomach extended caudally and ventrally, leading into the pylorus. The pylorus serves as a sphincter that connects the aboral part of the stomach to the anterior part of the small intestine. In two axolotls, it was positioned around the 7th costal groove.

The pylorus appeared as a circumferential thickening of the muscular layer, with a continuous wall connecting to both the stomach and intestine. Measurements of the pylorus diameter and wall thickness were taken from 16 axolotls, yielding a mean value of 1.76 mm (Table 3).

#### Small intestine and colon

Visualization of the small intestine proved challenging during ultrasound examination. In a single axolotl, we were able to observe the aboral part of the stomach and the oral part of the small intestine (Fig. 3B). However, the layered structure of the intestine could not be adequately discerned. Other portions of the intestine were not visualized at all. Nevertheless, we were able to illustrate the movement of the alimentary bolus between the stomach and intestine.

The colon was situated in the caudal region of the coelomic cavity, between the pelvic limbs, and continued caudally beyond the pelvic inlet. To visualize the colon, the probe needed to be positioned at the end of the coelomic cavity via a ventral approach. The ultrasonographic image of the colon revealed a barely distinguishable thin layered wall, with an underlying hyperechoic interface accompanied by its associated shadow cone artifact, indicative of intraluminal fecal content (Fig. 3C). However, visualizing the colon proved to be challenging and was achieved in only 4 axolotls.

#### Kidneys

The kidneys were observed in all axolotls, situated in the caudal region of the coelomic cavity. They were positioned in contact with the dorsal wall at the level of the pelvic limbs. In males, the kidneys were located caudally to the testes. Sagittal sections facilitated the measurement of length and height (h1), while the transverse view allowed for measurement of width and height (h2). The two measured heights (h1 and h2) were averaged to determine a single height (Table 3). The renal parenchyma exhibited a homogeneous appearance, being more hypoechoic than the margins, and delineated by a hyperechoic layer. Additionally, the renal parenchyma was found to be isoechoic to the epaxial muscles. A central anechoic vessel with a longitudinal axis was observed in both transverse and longitudinal sections.

#### Male reproductive tract

The testes are paired and symmetrical organs. However, they were not reliably found in all animals, with seven males having both testes observed and one male presenting with only one testicle. Positioned cranially to the kidneys, ventrally to the lungs, and sometimes reaching ventrally toward a part of the liver, the testes were best visualized using a dorsal paravertebral approach. It is recommended to first locate the kidneys and then advance the probe cranially to obtain longitudinal and transverse images of the testes. Occasionally, the lungs may obstruct their visualization due to reverberation artifacts. The testes appeared multi-lobed, hypoechoic compared to the liver, with homogeneous parenchyma. A very thin, hyperechoic wall surrounded them (Fig. 3G). Detailed measurements are provided in Table 3.

#### Female reproductive tract

The ovaries were poorly differentiated and consisted of multiple units, observed in all females. On ultrasonography, the ovaries appeared filled with follicles, manifested as several circular anechoic elements with millimetric hyperechoic elements inside (Fig. 3H). It was not possible to distinguish between the right and left ovaries. The oviducts were primarily located in the caudal and middle coelom and were prominent in all 15 sexually mature females. Arranged in organized loops, the oviducts exhibited a tubular appearance with a hyperechoic wall compared to the more hypoechoic inner tissue (Fig. 3I). Similarly to the ovaries, distinguishing between the right and left oviduct was not feasible.

#### Surface of the lungs

The lungs are paired, symmetrical, saccular organs, and the surface of both lungs was observed in all axolotls. Positioned dorsally in the cranial and middle coelom, the lungs were situated ventrally to the digestive organs, testes, and spleen, with the kidneys located caudally. The lung surface appeared highly hyperechoic, with reverberation artifacts, attributed to the presence of air, appearing ventrally along the entire lung surface.

#### Other observations

The skin appeared as a linear hyperechoic line superficial to the lungs. Ultrasonographic images of the spine displayed muscle appearances and rudimentary ribs. A small amount of anechoic fluid within the coelomic cavity was present in all axolotl. No wall was found to suggest the presence of a bladder. Deferent duct, ureters, adrenals glands, or pancreas were not visualized either.

## Discussion

Our primary objective was to establish an ultrasound protocol, provide confidence interval measurements of coelomic structures, and create an ultrasonographic atlas of the axolotl coelomic cavity (Supplement 2) for use in research or clinical settings. Post-mortem examination confirmed the localization and description of each major organ, enabling us to develop schematic drawings of the topographic anatomy of the coelomic organs (Supplement 1). A detailed description of the normal ultrasonographic appearance of coelomic organs is crucial for distinguishing abnormal from normal organs and enhancing our understanding of anomalies. Furthermore, it is pertinent to identify which organs are not typically visible via ultrasound.

Ultrasonography offers numerous advantages in animal species, including amphibians. It is a non-invasive, innocuous, and safely repeatable imaging modality.^18,33^ The ultrasonographic appearance of coelomic organs has been documented in amphibians in previous studies.^25,27^ However, there is limited literature describing axolotl ultrasonography specifically.^34,35^ A study on axolotls’ echocardiography is available, providing a detailed description of heart anatomy.^22^.

As axolotl health poses challenges, our primary was to monitor and measure our sample. Previous studies have reported average weights of 131.12 gr and 39.2 ± 25.2 gr, as well as body lengths of 24.6 cm and 17.2 ± 4.5 cm, respectively.^34,36^ Our population closely resembled these measurements; however, weights were slightly higher.

In consideration of animal welfare and ethical concerns, our aim was to devise a method that minimizes excessive manipulation of axolotls and avoids the use of anesthesia. Utilizing a probe with ultrasound gel would necessitate direct handling of the axolotls, with recommended rising of the skin after examination.^18^ However, due to the amphibian’s highly permeable tegument, which is easily damaged by handling and can lead to a loss of homeostasis,^37^ we do not recommended this method. The amphibian’s skin serves as one of the most vital organ systems, playing roles in respiration, defense against pathogens, thermoregulation, and hydration hemostasis, among others. It must always remain moist for effective gas exchange, making desiccation a constant threat to survival.^26,38^ Additionally, toxic effects of latex, vinyl, and nitrile gloves have been documented when working with amphibian. Therefore, it is recommended to rinse gloves in fresh water before use to remove talcum and reduce potential toxins.^39–41^.

Descriptions of ultrasonography performed directly in water, in small restrained spaces such as plastic bags or small plastic containers with anesthesia, are available and have shown satisfactory results.^18,22,25,42^ This approach allow us to avoid the use of general anesthesia, thereby reducing associated side effects. Anesthetized amphibians may experience respiratory depression, and recovery can be prolonged, necessitating close monitoring. Anesthesia in amphibians is documented with various agents such as tricaine methanesulfonate (MS-222), benzocaine sulfate, alfaxalone, propofol, and inhalant halogens (Isoflurane and Sevoflurane) administered via baths. However, doses must be carefully measured, and the duration of the examination should be considered to prevent prolonged recovery times for the animals.^43^ The restraint method used in our study can also induce stress or discomfort, as evidenced by increased heart rate and movements such as half-turn of axolotls. To mitigate these undesirable effects, an operator can use slight sedation (e.g., 5 mg/L of alfaxalone in a water bath) and reduce the procedure time as much as possible.^44^.

After initial attempts, it took approximately 15–20 min to complete a full ultrasonographic examination. The complete protocol was performed by all three operators in the same order without difficulty, ensuring uniformity. Ultrasonography using a 7- to 15-MHz linear array transducer enabled visualization of the following coelomic structures in axolotls: heart, lung surface, liver, gallbladder, spleen, stomach, pylorus, testes, genital tract, and kidneys. However, small organs such as the pancreas or adrenal glands were not detected in our study. This could be attributed to their small size and resolution limitations. Higher frequency transducers (20–25 MHz) are likely to provide better data in axolotls due to their enhanced image quality.

These data enabled us to establish measurements for normal organs, which could then be utilized for comparison with abnormal organs. Topography plays a crucial role in ultrasound imaging, and in this context, we considered it relevant to count axolotl costal grooves to assist future operators in localizing coelomic organs. However, these counts were challenging to obtain and feasible only in light animals.

The heart was easily identifiable in all individuals, exhibiting continuous contractions associated with blood movement and surrounded by a pericardial sac. Similar to previous ultrasonographic examinations in amphibians, it served as a reliable landmark to initiate the examination.^25,26^ However, compared to the study by Dittrich et al*.,* our images were less precise. To enhance image magnification, we utilized 50 MHz probes for axolotls weighing less than 20 gr and 40 MHz for those weighing more than 20 gr.^22^ In contrast, we conducted examinations on animals with an average weight of 69.19 ± 31.06 gr using a 15 MHz frequency device. The placement of the transducer followed Dittrich et al*.*’s method to allow for comparison.^22^ Thus, images capturing the ventricle long axis, atria long axis, midline long axis, and ventricle short axis were obtained. However, the choice of the midline long axis remains unclear in this study, as its placement was not strictly median and close to the atria long axis. Unlike in the study by Dittrich et al*.*, the oblique paragill view was not performed as it did not provide significant information. Despite some movements exhibited by axolotls, the repeatability of these views was assessed even without anesthesia. It is important to note that anesthesia can slow heart rate, which must be taken in account when designing experiments.^43^.

Ultrasound frequencies between 7 and 15 MHz were found to be suitable for measuring heart rate in axolotls (45.33 ± 11.7 bpm). In comparison, the resting conscious heart rate measured using Eulerian video magnification was reported to be 21.7 ± 4.1 BPM, which is lower than our data.^36^ However, Eulerian video magnification allows for data collection without manipulation. Another study describes a mean heart rate at of 22.23 ± 5.18 BPM at rest and a mean heart rate of 39.44 ± 2.38 BPM during exercise, which correlates with our data.^45^ This increased heart rate during examination could be attributed to movements associated with restraint or stress, as previously demonstrated in amphibians.^46,47^ Therefore, we strongly recommend heart rate at the beginning of an experiment or clinical examination.

The lung surface appeared highly hyperechoic with reverberation artifacts, attributed to the presence of air ventrally on the lung surface. Relevant publications on amphibian lung appearance are scarce, with only one reported case of bilateral pneumonia in African clawed frogs (*Xenopus laevis)* available*.*^48^ In this case, one frog lung displayed severe pneumonia with parenchymal consolidation of the left lung observed on ultrasound, while the other lung maintained normal reverberation artifacts and displayed a B line which is a discrete, laser-like, vertical, hyperechoic image. These findings were consistent with fluid within the central airway of both frog lungs.

In our study, B lines were not observed, and reverberation artifacts were found throughout every axolotl’s parenchyma. Considering that anurans and urodeles may present anatomical differences, it remains unclear whether the presence of B lines is always pathological in different amphibian species.

Our protocol recommends scanning the liver using a ventral approach caudally to the heart, which is similar to techniques used in other amphibians.^25^ The hepatic parenchyma appeared homogeneous, and the liver had sharp and regular edges. Unlike some other species, the axolotl liver does not exhibit an apparent lobe structure. Although lobe-like clefts exist, they are identifiable only though correlative microscopy and block-face imaging.^49^ Consequently, liver lobes could not be differentiated by ultrasonography. The vascularization of the liver typically included a major right hepatic vein originating from the inferior vena cava, which divides into two left hepatic veins.^46^ However, in our study, only the caudal vena cava, leading to the heart, was observed by ultrasound in all axolotls. This vessel was characterized by a tubular structure with hyperechoic walls and hypoechoic content.

Like in other amphibians, the gallbladder in axolotls was associated with the liver parenchyma and appeared as an anechoic oval structure with a hyperechoic wall on ultrasonography.^19,47^ Although the axolotl’s gallbladder wall is composed of the mucosa, muscularis, perimuscular, and serosa, theses layers were not observed on ultrasound images.^24^ Hyperechoic millimetric particles were found in the lumen of the gallbladder in nine axolotls, which is consistent with images obtained in the olm (*Proteus anguinus)* and in four axolotls in another study*.*^35,46^ Larger hyperechoic particles have been reported in one axolotl and in cases of cholelithiasis in the giant ditch frog (*Leptodactylus fallax*).^18,50^ As our axolotls display a normal clinical state, it remains difficult to determine whether the presence of hyperechoic millimetric particles in the gallbladder is physiological or pathological. In companion animals, other ultrasonographic signs of cholecystitis and cholelithiasis may include thickening or irregularity of the gallbladder wall, as well as gallbladder distension. Thickening and irregularity are important to verify, as they can indicate cholecystitis in companion animals.^51^ Gallbladder distension is also recognized as an ultrasonographic feature in cholelithiasis and cholecystitis. However physiological variations in gallbladder dimensions have been reported in olms and in mammals.^46,52^ According to the organ’s axis, this parameter showed significant differences; therefore, it was not measured in this study.

The spleen was situated on the left visceral surface of the liver and was observed in all individuals. In this study, the splenic parenchyma was found to be isoechoic to hypoechoic compared to the hepatic parenchyma, which is consistent with the findings of another study.^35^ The contact surface between the liver and spleen was small, making their differentiation difficult.

The digestive system appeared easily visualizable in all axolotls. The histology of the stomach consists of four layers: mucosa, submucosa, muscular layer, and serosal layer.^26^ While the mucosa, submucosa, and muscular layer were observed and measured on 20 out of 28 axolotls, several measurements of the stomach wall were hindered by gastric content. Although we were unable to observe the serosa of the stomach, it was identified in a recent report.^35^ Fasting for 24 h is described to avoid regurgitation in case of anesthesia; however, the fasting period was arbitrarily suggested to be 72 h.^53^ Yet, it was not possible to prevent the axolotls from eating their own eggs or feces, which precluded proper ultrasonography examination. A 7-day fast can be performed to avoid gastric elements, without manifestly affecting the health of the animals.^35^.

As in mammals, the pylorus was formed with a thicker muscular layer.^24,26^ Although the neotenic intestinal tract was not observed in our study, it is described as atypical vertebrate intestines, without a valve separating the small intestine from the large intestine. In addition, an ultrasound research on 11 axolotls also failed to observe the intestinal tract.^35^.

The renal parenchyma was found to be isoechoic to the epaxial muscles and delimited with a hyperechoic layer. Paired kidneys were situated dorsally at the caudal extremity of the coelomic cavity. In the literature, they are described as “pear-shaped” organs close to each other with a narrow anterior and wide posterior. the axolotl kidney’s basic unit is the nephron, similar to mammals, which is composed of a glomerulus and a tubule system.^54^ Similarly to *Xenopus laevis,* the kidneys were better identified using the dorsal approach.^55^ Other amphibians’ kidneys (*Proteus anguinus, Andrias davidianus* and *Dyscophus antongil)* are located in the caudal part of the coelomic cavity.^25,46,56^ In axolotls, they were easily observed dorsally to the pelvic members. A central vessel was observed in all axolotls. The vascularization of the axolotl’s kidney is identical to other urodeles, with blood supply to the ventral surface consisting of renal arteries from the dorsal aorta and efferent renal veins joining the caudal vena cava which runs along the ventral surface of each kidney.^57,58^.

In this study, the urinary bladder was not identified via ultrasonography within the allocated time or during necropsies, contrary to previous observations in adult salamanders as reported by Wright et al.^18^ Textbook descriptions suggest that the urinary bladder appears as a smooth-surfaced, spherical, anechoic structure in the caudal abdomen, although some salamander species may possess a cylinder-like or bilobate bladder.^26^ Another article mentions obtaining urine samples from the bladder in *Ambystoma mexicanum* cloaca, albeit without precise anatomic description. Additionally, a study on the ultrasound description of the coelomic cavity of axolotls also reports not finding the bladder.^35^ To confirm the localization of the urinary bladder, contrast studies with computed tomography or cloacoscopy would have been interesting approaches.^59^.

Ovaries were readily identifiable, displaying a plethora of follicles in all female axolotls, akin to observations in other amphibian species such as the Madagascar tomato frog (*Dyscophus antongil*) or the banded bullfrog (*Kaloula pulchra*).^25,55^ These follicles appeared as millimeter-sized rounded anechoic structures with hyperechoic speckles surrounded by a hyperechoic layer. In amphibians, gonads typically reside cranially to the kidneys. While follicle maturation is precisely described in anurans (*Necturus spp.),* we were unable to discern the exact stage of follicular maturation in our females using our ultrasound frequencies.^29^ Due to the substantial size of the ovaries, it was not always feasible to laterally differentiate the follicles, although this distinction is possible in some anuran species.^55^.

Near the ovaries, convoluted and coarse oviducts were observed, appearing slightly hyperechoic compared to the liver. Macroscopically, the oviducts presented as two elongated, white, convoluted structures, occupying significant space.^60^ In comparison to other amphibians, the oviducts in axolotls were notably developed.^25,26,29^ They exhibited a hyperechoic wall with inner hypoechoic tissue. The absence of distinct layer structure and peristaltic activity aided in distinguishing the genital tract from the digestive tract.

While ovaries and oviducts were readily observed, testes were not consistently identified. They were primarily located cranially to the kidneys above the pelvic members, using a dorsal approach. The testes appeared as multi-lobed structures, hypoechoic compared to the liver, with homogeneous parenchyma. In urodele amphibians, testes typically manifest as elongated bilateral structures. In adult specimens, their morphology can vary significantly across clades, ranging from single elongated structures to lobed configurations attached by connective tissue. In *Ambystoma mexicanum*, for instance, each testis comprises numerous lobes, which may exhibit distinct separations externally, delineated by constrictions and differences in coloration.^60–62^ Surprisingly, our ultrasonographic images did not reveal clear separation between lobes but displayed homogenous content. Adjacent to the testes are the paired fat bodies, whose size varies among axolotls. Fat bodies can range from absent to thin or very large, which may account for their non-observation in contrast to findings in another study.^35,60^.

All axolotls exhibited a small quantity of free anechoic fluid surrounding the organs in the coelomic cavity. This is a common finding in amphibians and should be considered physiological, distinct from hydrocoelom (ascites). Large amounts of fluid are deemed abnormal when they fulfill the coelomic cavity, causing the liver or gastrointestinal tract to float within the fluid.^26^.

The primary limitation of this study was the small sample size of 28 animals, with 25 animals originating from the same breeder. Given that some axolotls were genetically related, this could introduce bias into the results. Additionally, the health status of the animals was solely assessed clinically. While a blood test would have been informative to confirm the state of health, we opted for a non-invasive approach to minimize animal stress and avoid owner refusal. Furthermore, the reference values available were obtained from anaesthetized animals, which differs from our study conditions.^63^.

Our findings corroborate those of a previous study conducted on 11 live axolotls, albeit on a larger sample size. While the protocols and measurement methods differed between the two studies, our results offer a valuable complement to the existing data. It is worth noting that in the previous study, several measures were taken on each animal, but the statistical method employed did not adequately account for these repeated measures, potentially resulting in an underestimation of the true variation.^35^ The larger sample size in our study allowed us to establish confidence intervals for the 28 individuals included, although these intervals should be considered when interpreting the results or comparing them to other axolotls. Combining the measurements from both studies on a larger sample would be beneficial for obtaining reliable standards for this species.

Our results were also constrained by the resolution limitations of the highest frequency linear transducer (7–15 MHz) currently available in veterinary clinics, which hindered the visualization of certain coelomic organs. Despite this limitation, the protocol was successfully executed by three different operators, demonstrating its repeatability and ease of implementation.

In conclusion, this study describes the ultrasonographic characteristics of coelomic organs in axolotls. Ultrasonography emerges as a safe and easily executable procedure in this species. The established protocol facilitates the clear visualization of major coelomic organs with minimal stress for axolotls. The accompanying atlas and schematic drawings serve as valuable resources for comprehending organ topography and conducting examinations on other animals. Nevertheless, uncertainties persist regarding the ultrasounds localization of the bladder in this species and the nature of hyperechoic particles in the gallbladder, necessitating further investigation.

### Supplementary Information

Supplementary Information 1.

Supplementary Information 2.

## Data Availability

The data presented in this study are available upon request from the corresponding author.
