# Supplementary material for: Ultrasound description of the coelomic cavity of the axolotl (Ambystoma mexicanum) in a clinically healthy population: a pilot study
Source: Sci Rep. 2024 May 23;14:11787. doi: 10.1038/s41598-024-62264-z (PMC11116527; doi:10.1038/s41598-024-62264-z)
Supplement: Supplementary file 1 — Supplementary Information 1. [file 41598_2024_62264_MOESM1_ESM.pdf]

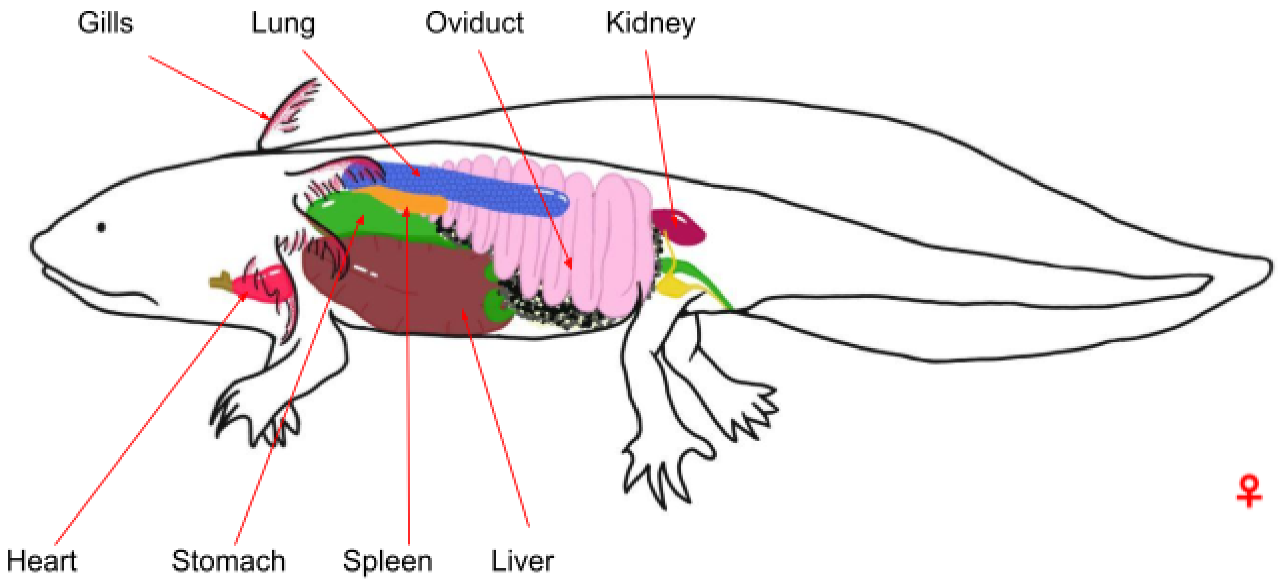

Left lateral view of the coelomic cavity in female axolotl.

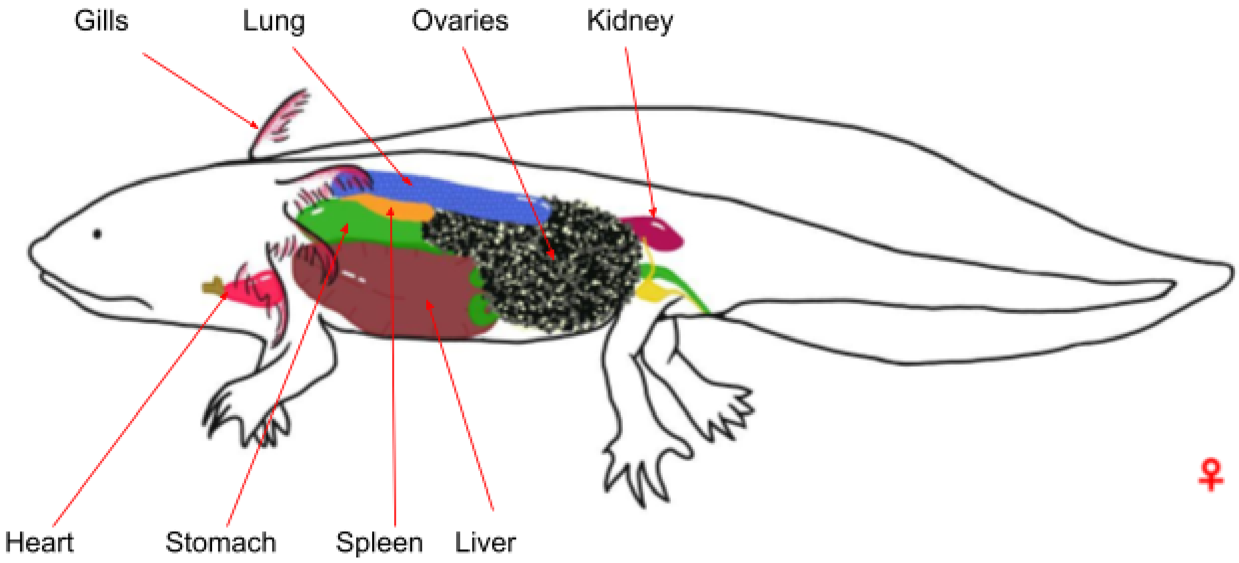

Left lateral view of the coelomic cavity in female axolotl without oviduct

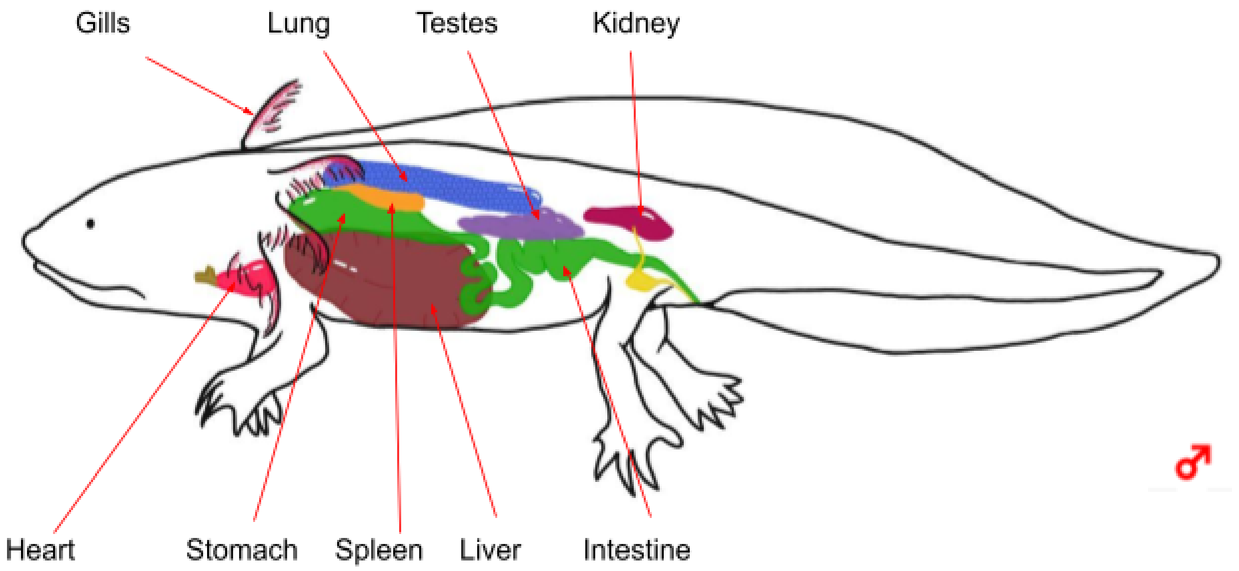

Left lateral view of the coelomic cavity in male axolotl.

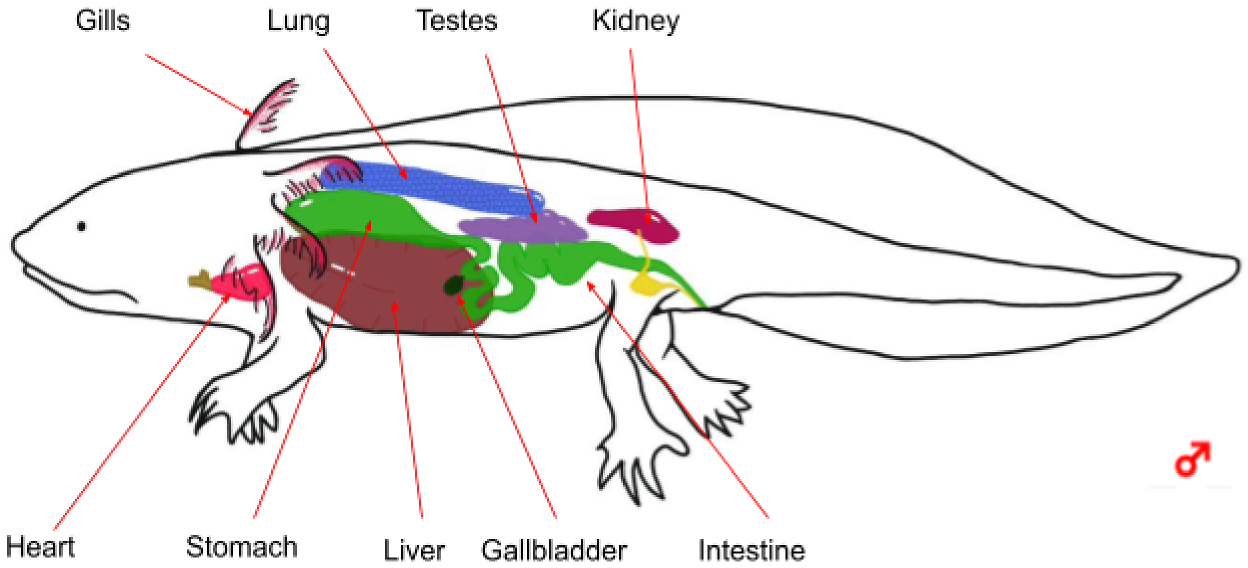

Right lateral view of the coelomic cavity in male axolotl.

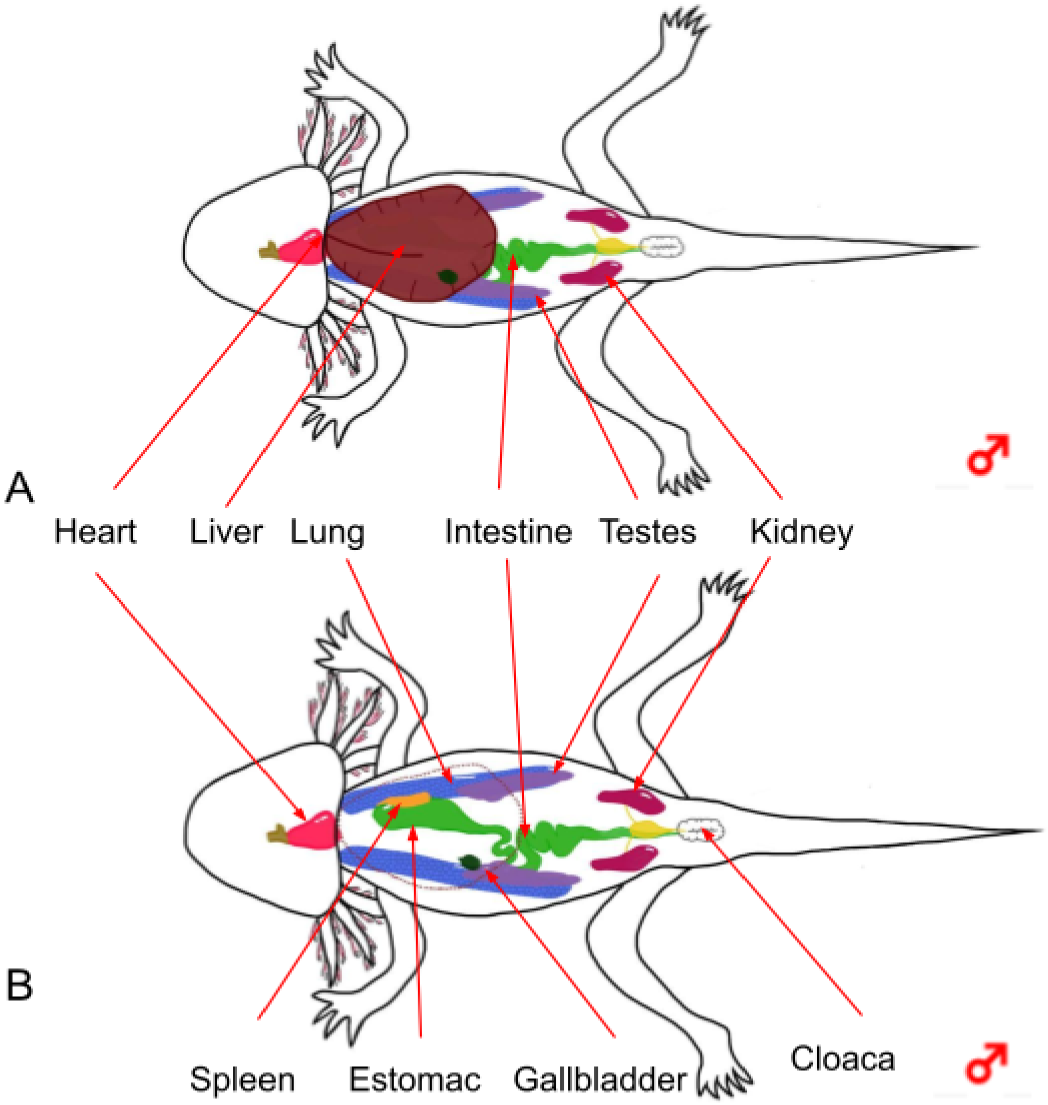

A: Ventral view of coelomic cavity in male axolotl. **B** : ventral view in male axolotl with liver removed.

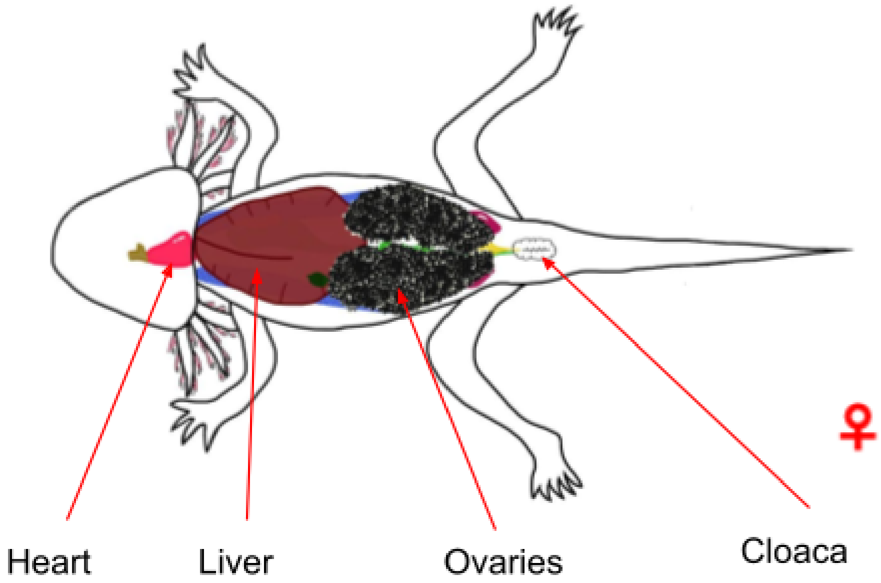

Ventral view of coelomic cavity in female axolotl with oviducts removed.
