# Supplementary material for: Ultrasound description of the coelomic cavity of the axolotl (Ambystoma mexicanum) in a clinically healthy population: a pilot study
Source: Sci Rep. 2024 May 23;14:11787. doi: 10.1038/s41598-024-62264-z (PMC11116527; doi:10.1038/s41598-024-62264-z)
Supplement: Supplementary file 2 — Supplementary Information 2. [file 41598_2024_62264_MOESM2_ESM.pdf]

Ultrasonographic atlas of axolotl coelomic cavity

In all figures, the pink probe represents longitudinal sections and the green probe represents transverse sections. The ultrasound protocol begins with the organs visible from the ventral view, in craniocaudal order. Next, the organs visible from the dorsal view are discussed, also in craniocaudal order.

Orientation is indicated with abbreviations : **Cr** = Cranial, **Cdl** = Caudal, **Vt** = Ventral, **Ds** = Dorsal, **L** = Left, **R** = Right.

VENTRAL APPROACH

Heart

Probe placed ventrally at the base of the neck

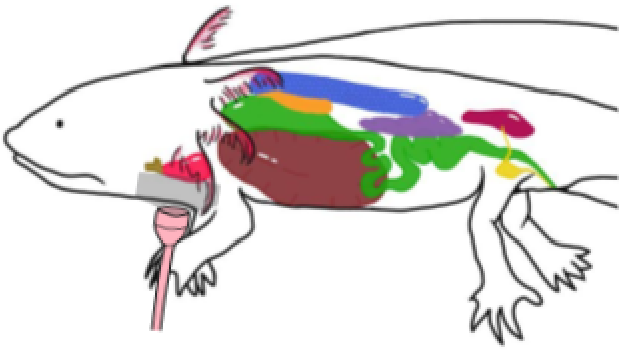

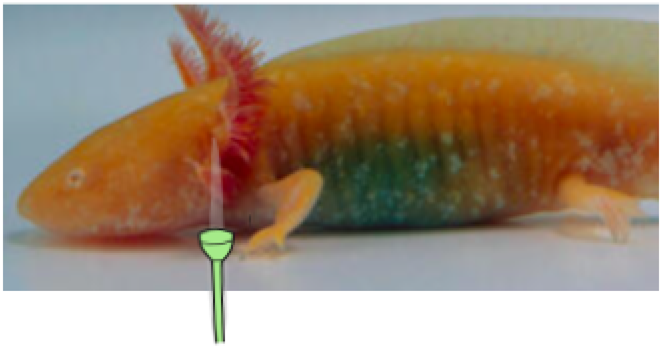

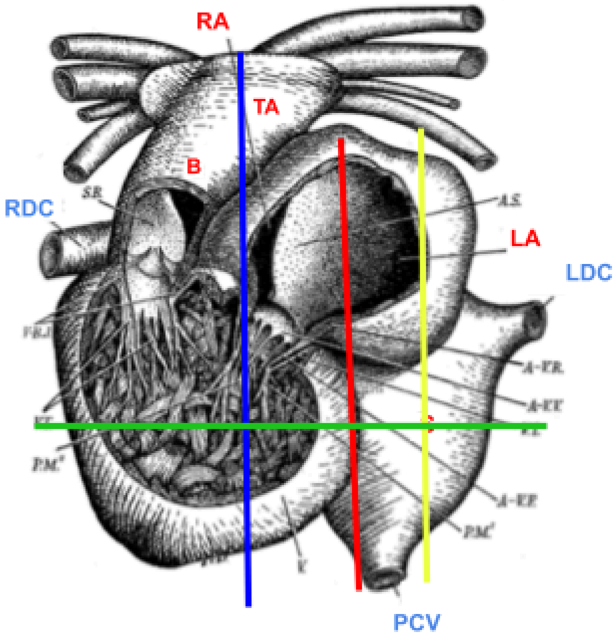

A : Anatomical drawing of the salamander heart, *Davies F. 1941*
RDC = Right duct of Cuvier , **LDC** = Left duct of Cuvier, **PCV** = Post-caval vein, **B** = bulbus cordis, **S** = sinus, **TA** = aortic trunk, **RA** = Right atrium, **LA** = Left atrium.
Yellow line = midline long axis view. **Blue line** = ventricle long axis view. **Red line** = atria long axis view.
Green line = short axis

☐ Long axis, midline view

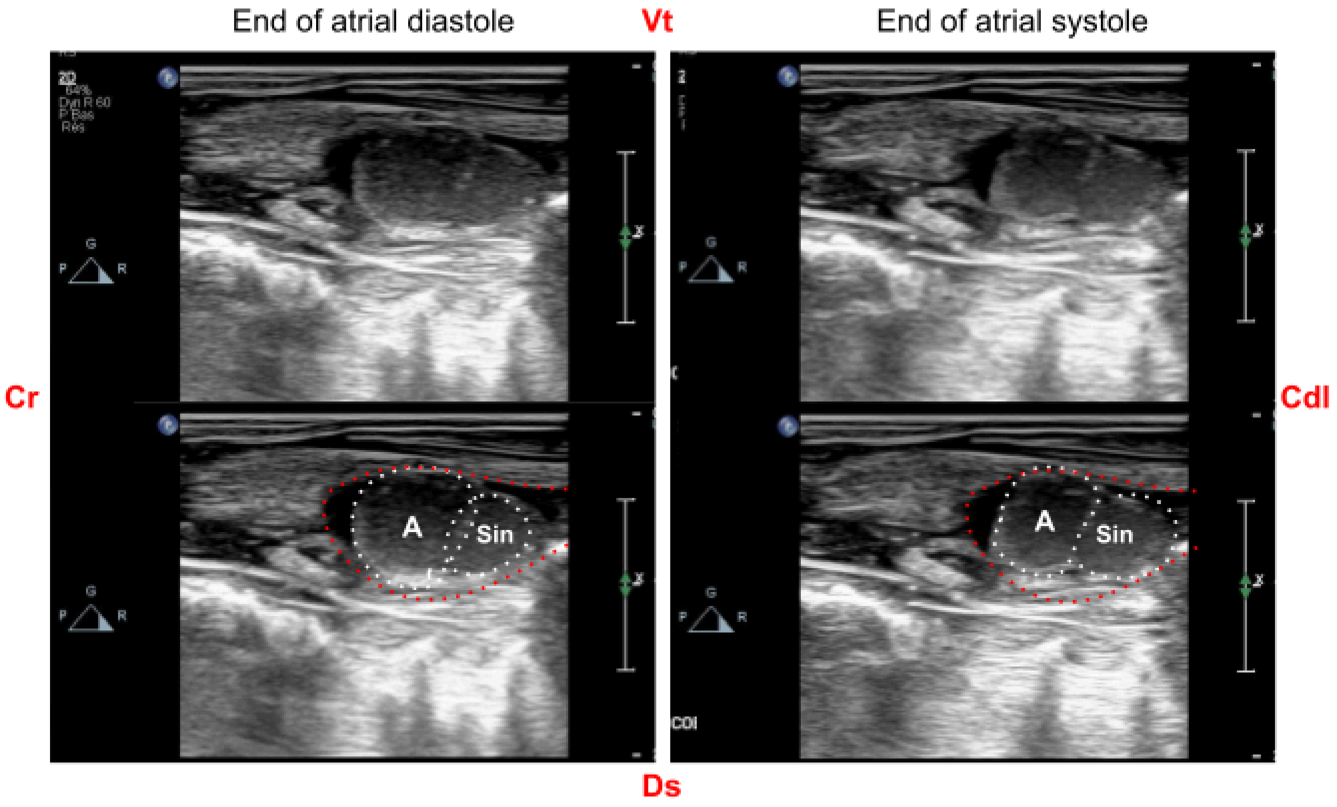

A = Atria (left and right atrium), **Sin** = Sinus, **Red dot lines** = Pericardium with physiological pericardial effusion

☐ Long axis, ventricle view

Probe moved to the right from the midline view

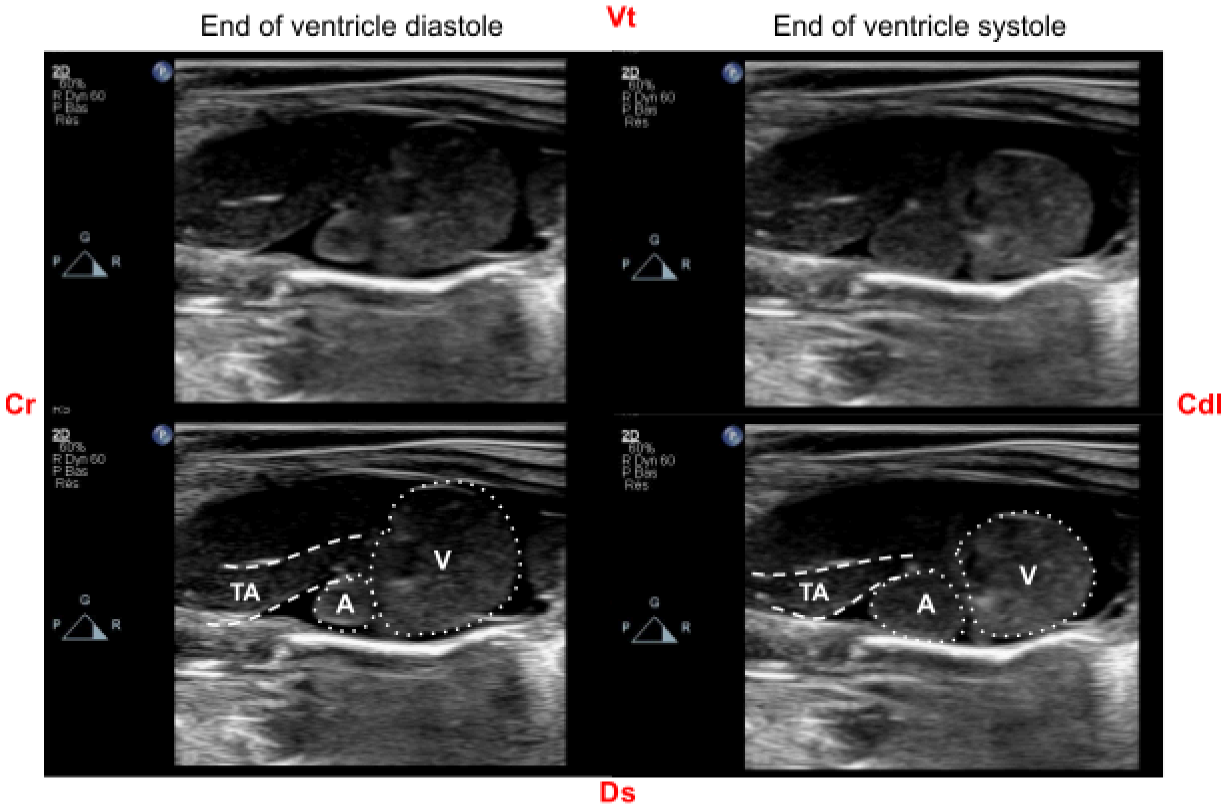

A = Atria (left and right atrium), **V** = Ventricle, **TA** = Aortic trunk

☐ Long axis, atriums

Probe moved slightly to the left of the ventricle long axis view

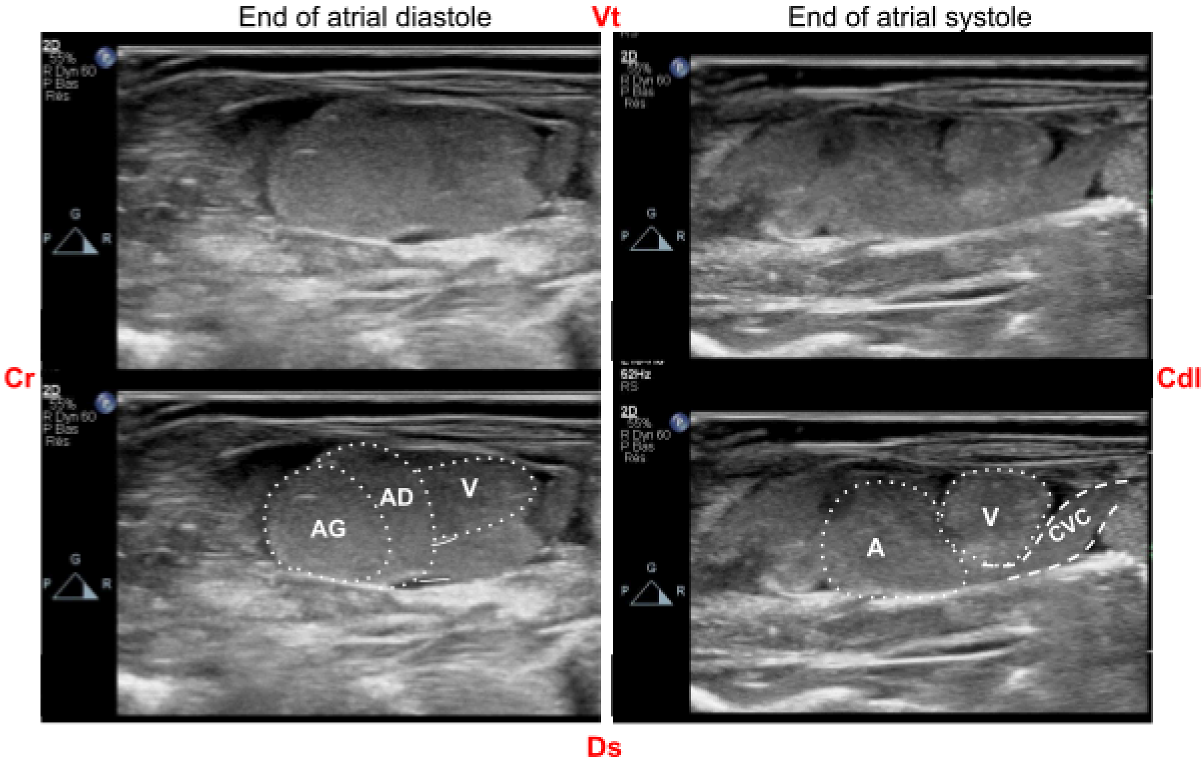

A = Atria (**LA** = Left atrium, et **RA** = Right atrium), **V** = Ventricle, **CVC** = Caudal vena cava

☐ Short axis, ventricle

Probe rotated 90° from long axis view

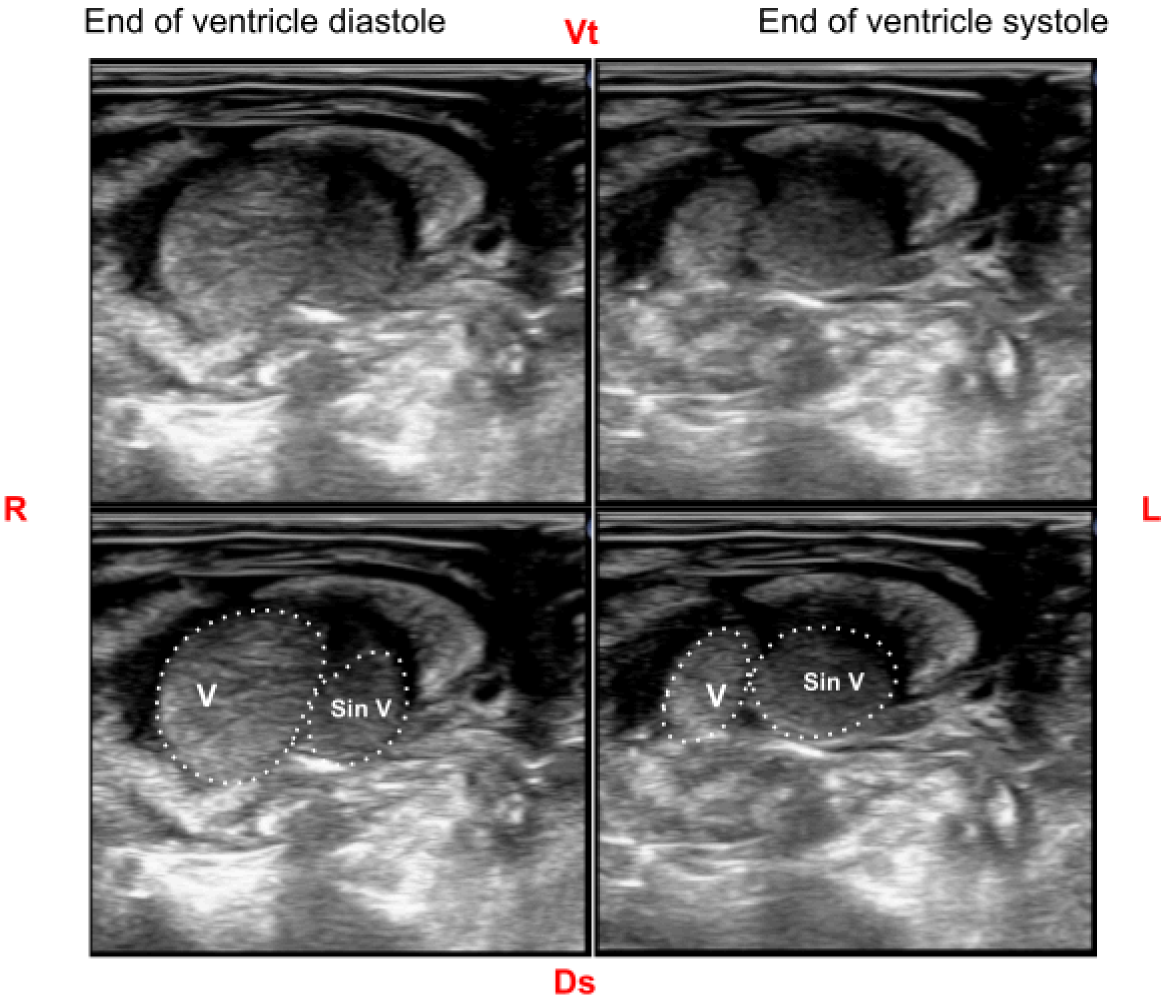

[illegible]

Liver

Probe placed ventrally, caudally to the heart

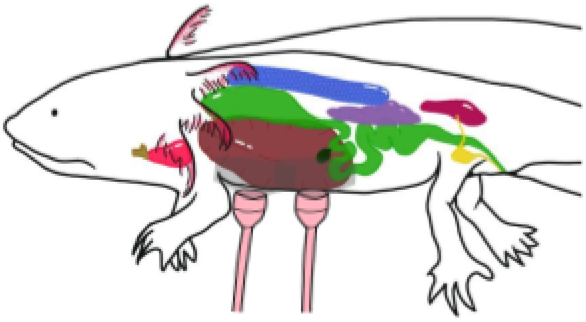

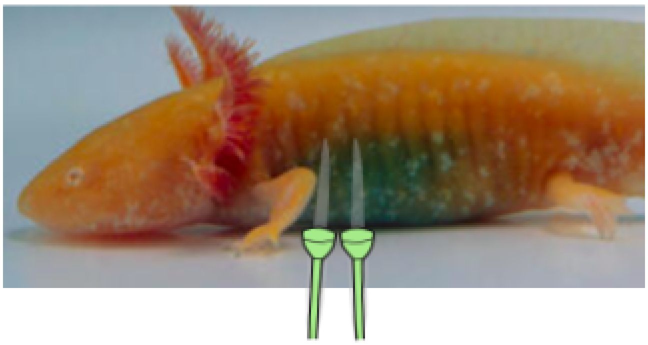

☐ Liver long axis

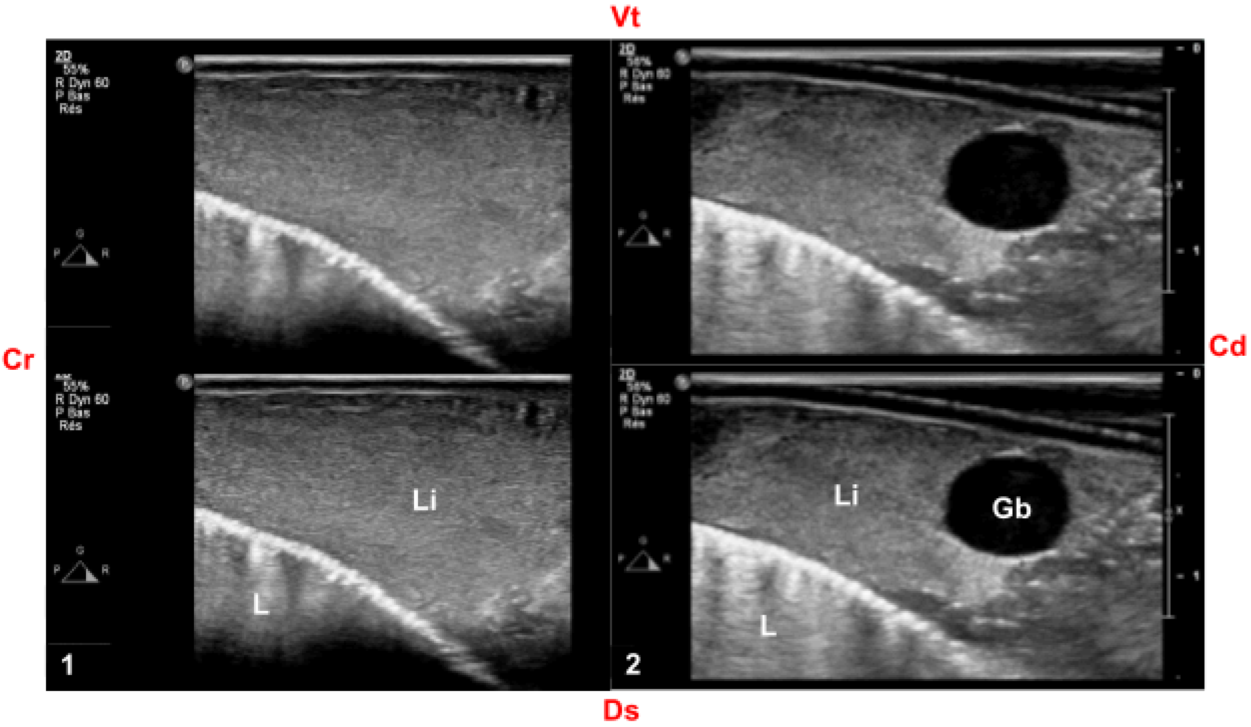

1 = Cranial liver. **2** = Caudal liver. **Li** = Liver, **Gb** = Gallbladder, **L** = Lung

- ☐ Liver short axis

Probe rotated 90° from longitudinal section

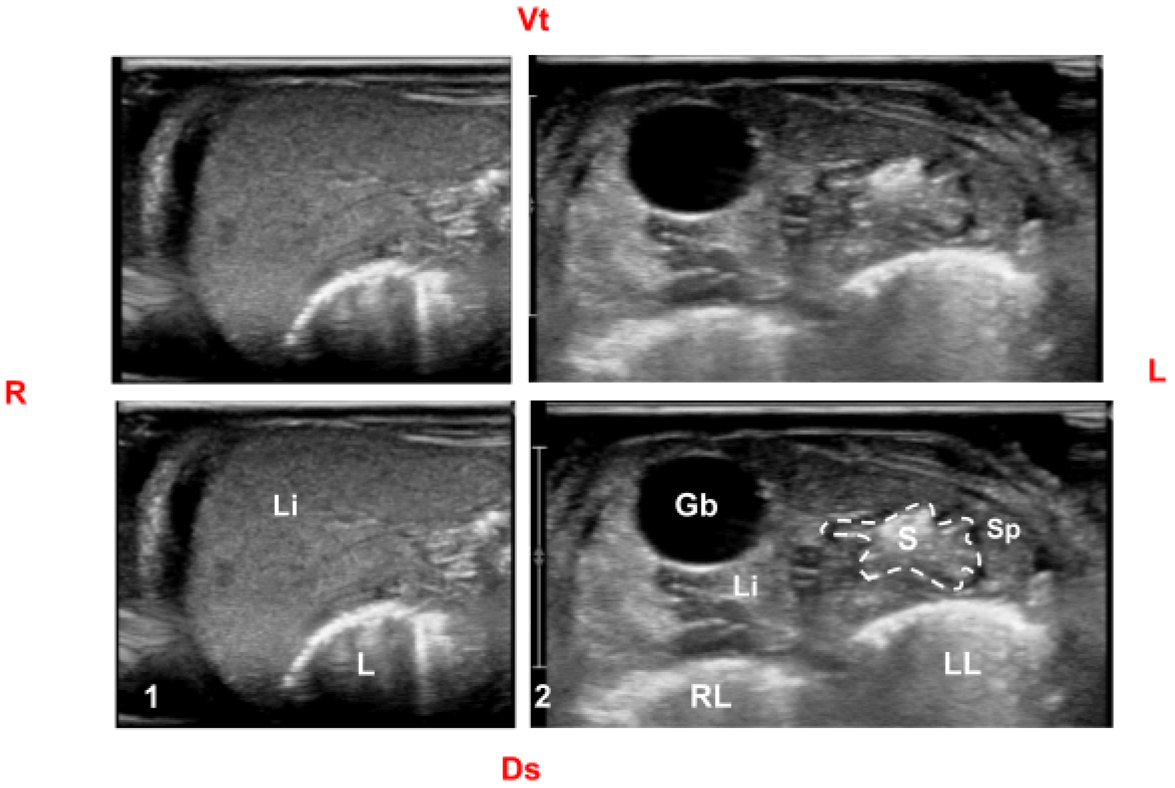

1 : Cranial liver. **2** : Caudal liver. **Li** = Liver, **L** = Lung (LL = Left lung, **RL** = Right lung), **Sp** = Spleen, **Gb** = Gallbladder, **S** = Stomach

Spleen

Probe placed ventrally, the spleen is located on the left in the coelomic cavity.

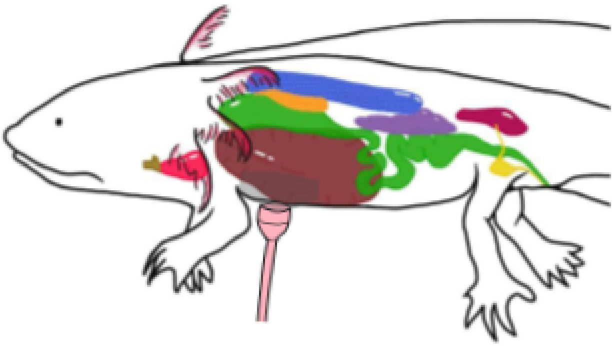

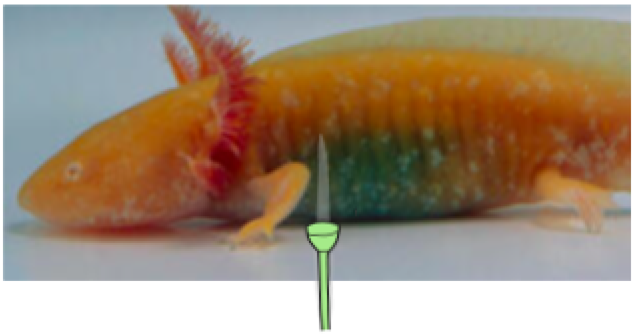

☐ Spleen long axis

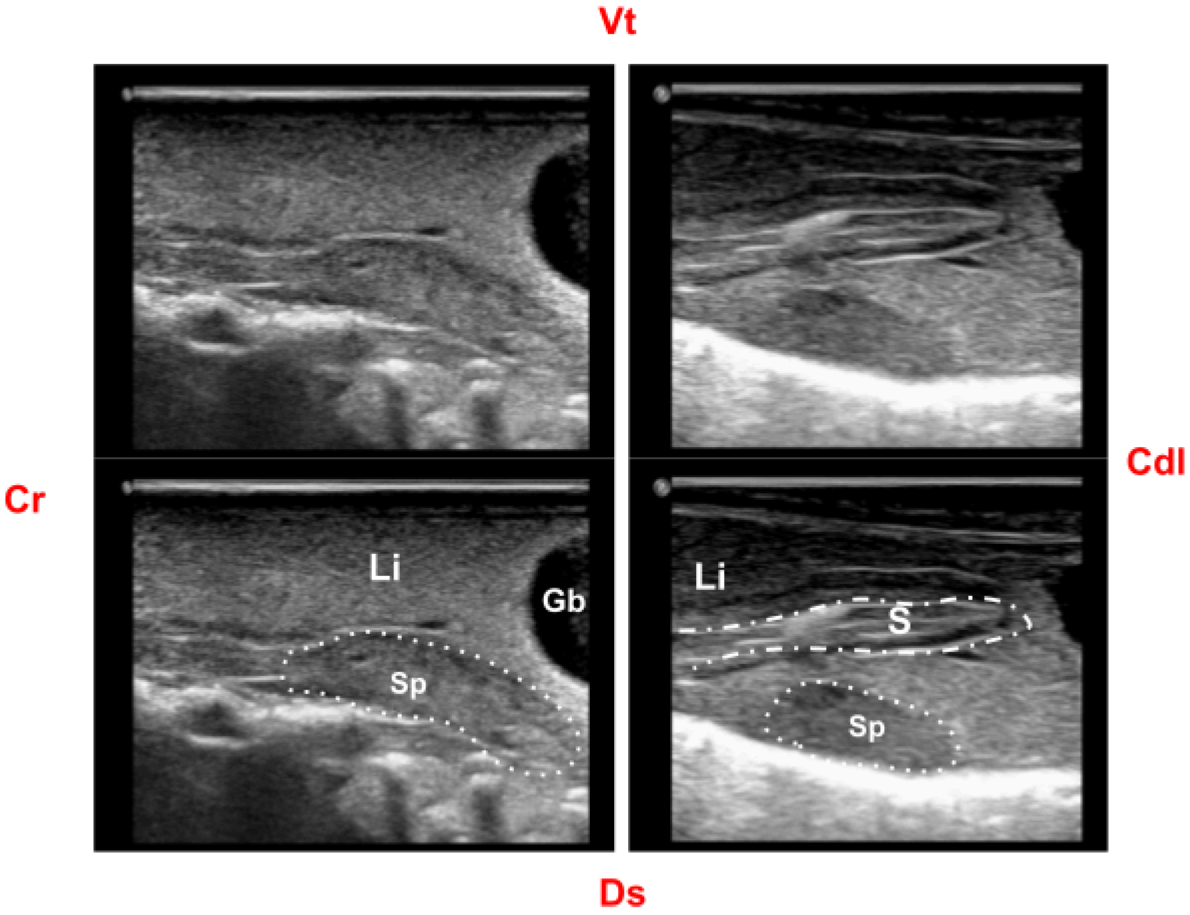

Sp = Spleen, **Gb** = Gallbladder, **Li** = Liver, **S** = Stomach

☐ Spleen short axis

Probe positioned at 90° to the longitudinal section of the spleen

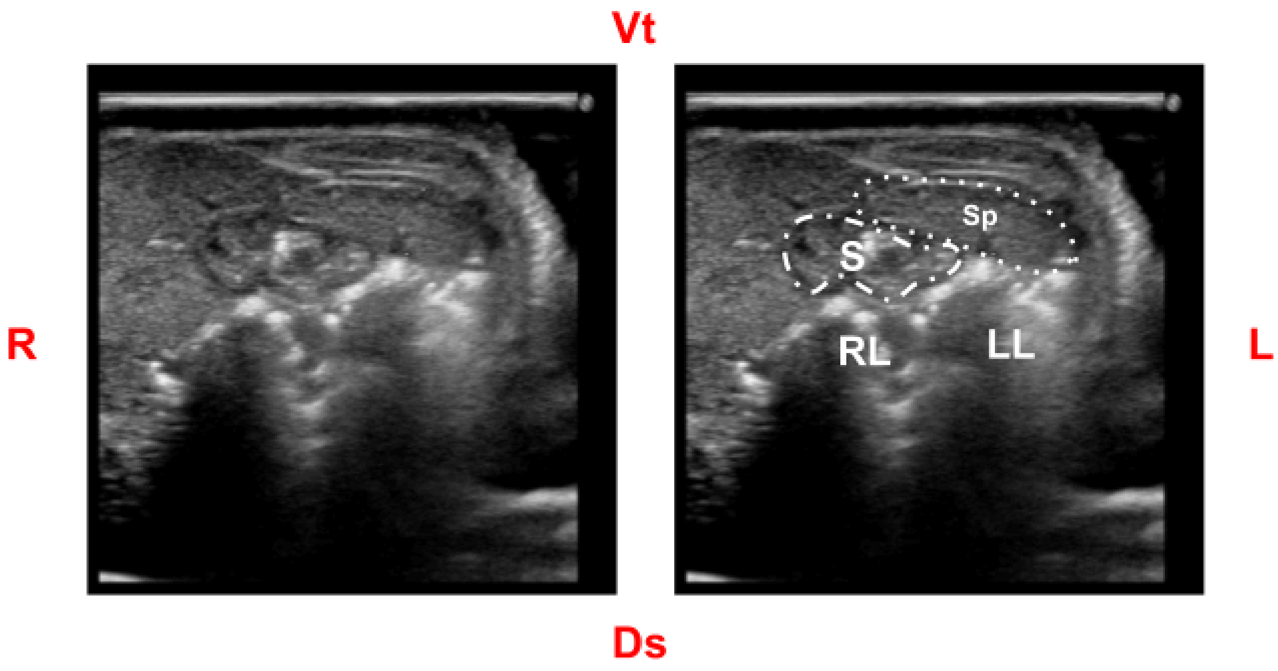

Sp = Spleen, **S**= Stomach, **LL** = Left lung, **RL** = Right lung

Stomach

Probe placed ventrally, stomach size and position vary according to its state of replenishment. The stomach is located to the left of the liver. The structure of the empty stomach is shown on the caudal cross-section of the liver.

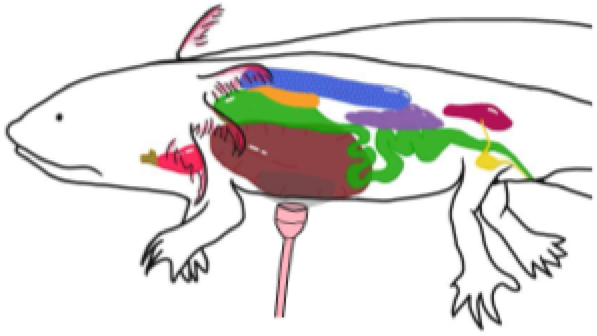

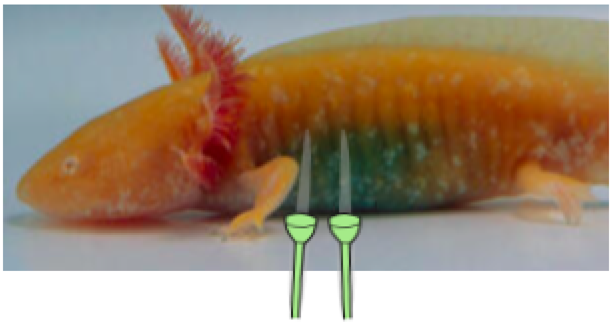

☐ Long axis, full stomach

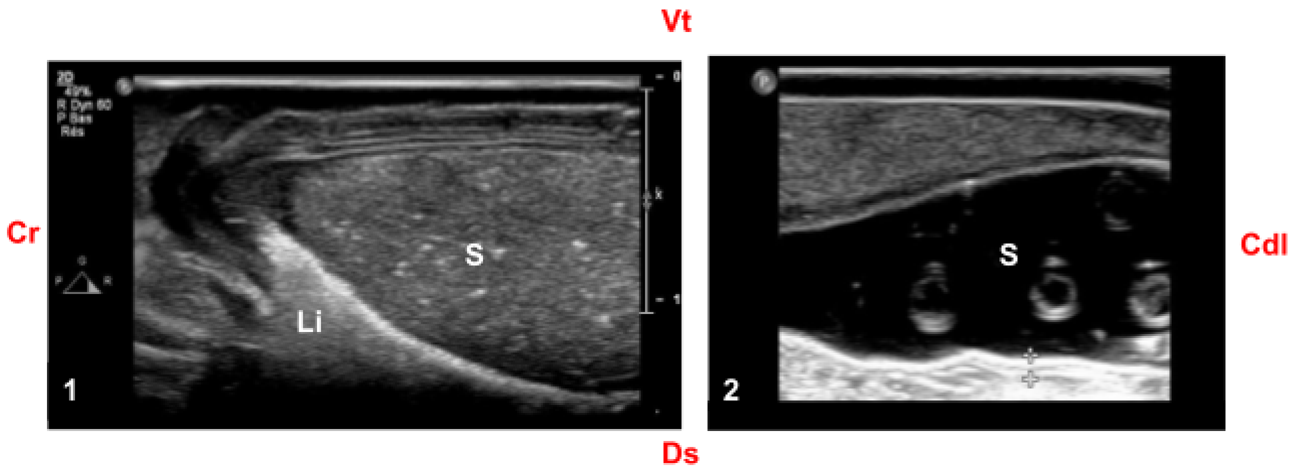

1 = Stomach content . **2** = Eggs in stomach
S = Stomach, **Li** = Liver

☐ Short axis, full stomach

Probe positioned at 90° to the longitudinal section of the stomach

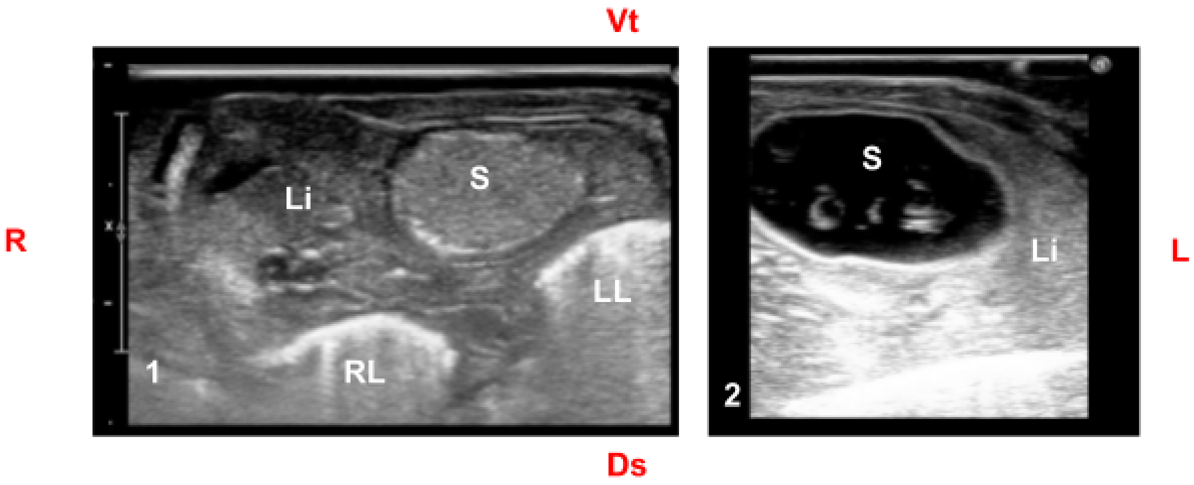

1 = Stomach content **2** = Eggs in stomach
S = Stomach, **L** = Liver, **LL** = Left lung, **RL** = Right lung

Pylorus

Probe placed ventrally, caudally to the stomach. The pyloric wall is continuous with the gastric wall.

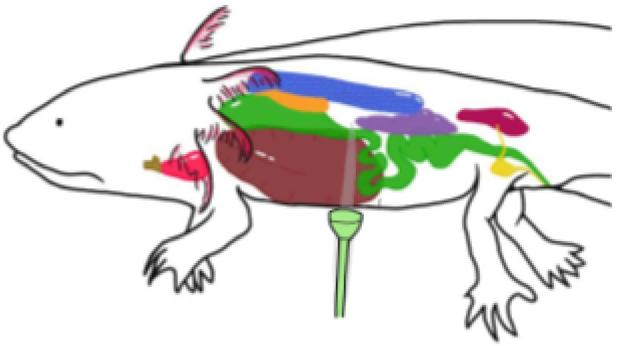

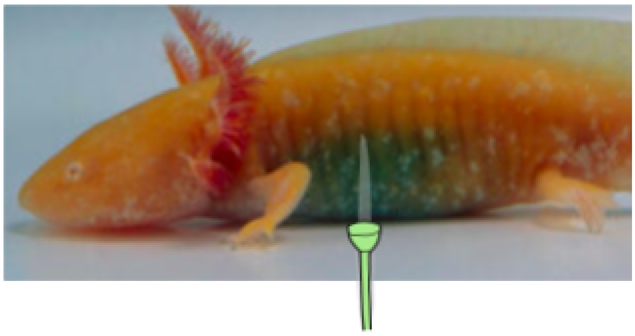

☐ Pylorus short axis

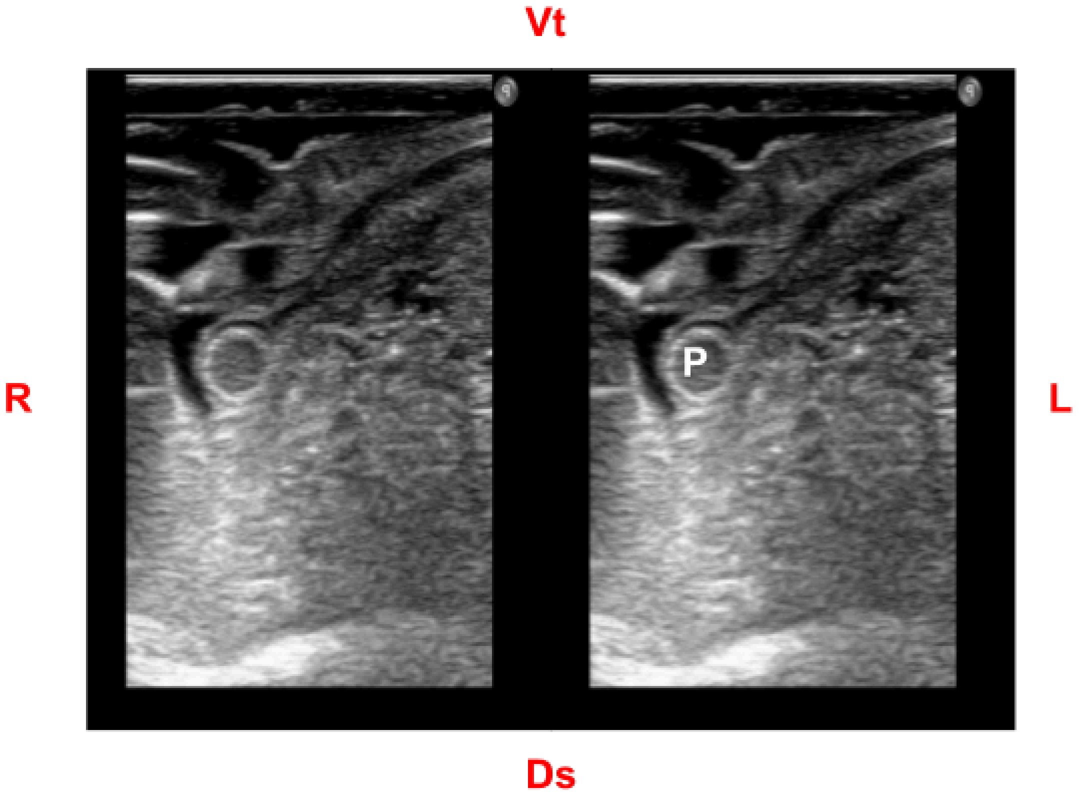

P = Pylorus

Intestine

Probe placed ventrally, caudally to the stomach. The small intestine was visualized in longitudinal section in a single axolotl.

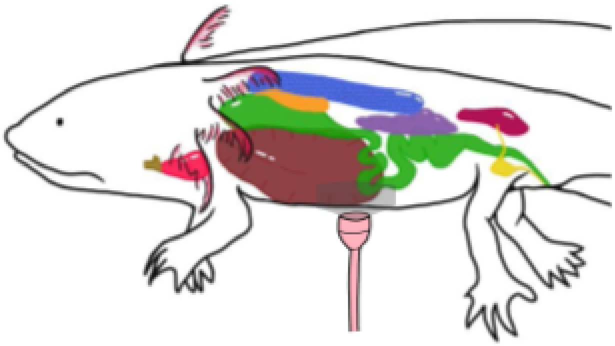

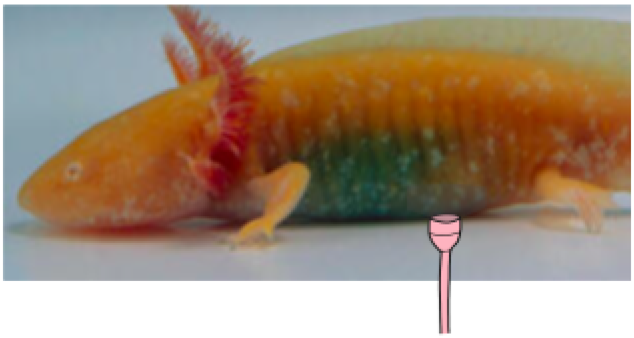

☐ Intestine long axis

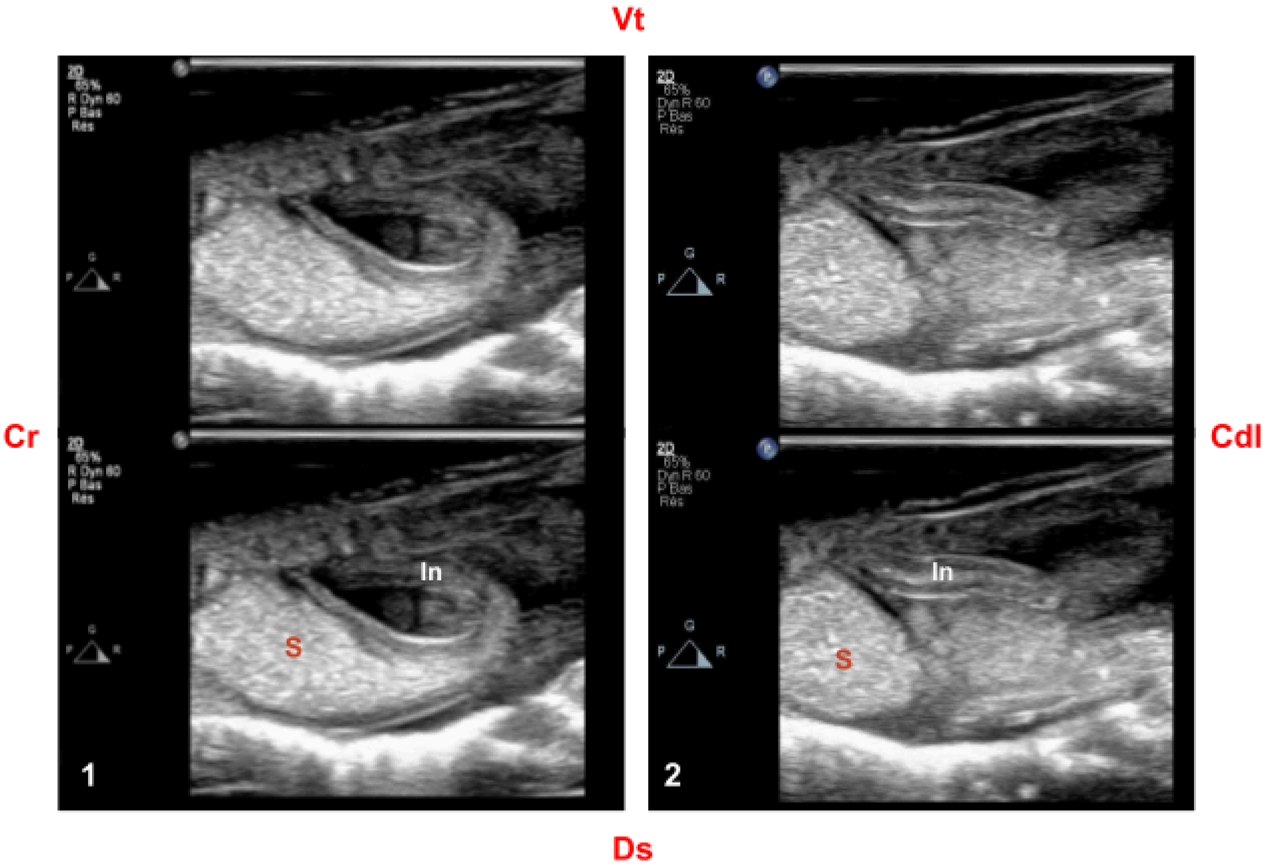

View 2 follows intestine loop. **S** = Stomach, **In** = Intestine

Colon

Probe placed ventrally, cranial to pelvic limbs

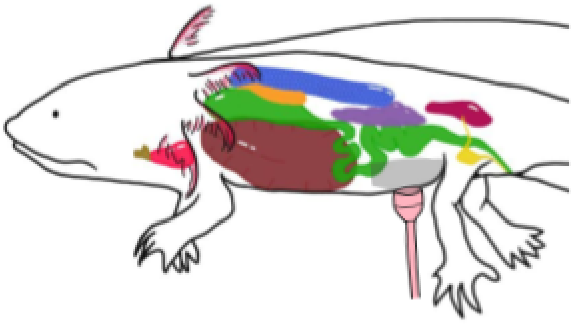

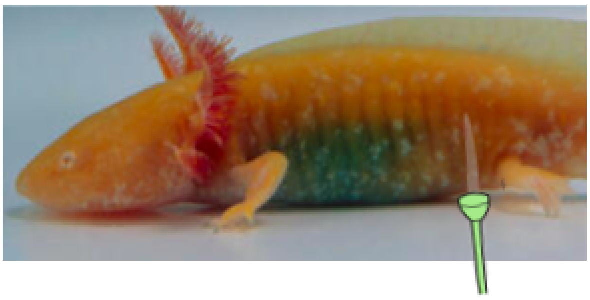

☐ Colon long axis

Vt

Cr 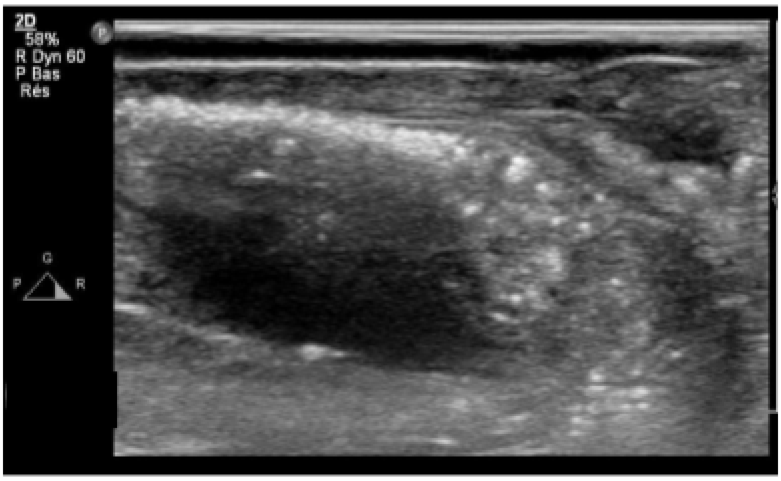 Cdl

Ds

☐ Colon short axis

Probe positioned at 90° to the longitudinal section of the colon

Vt

L 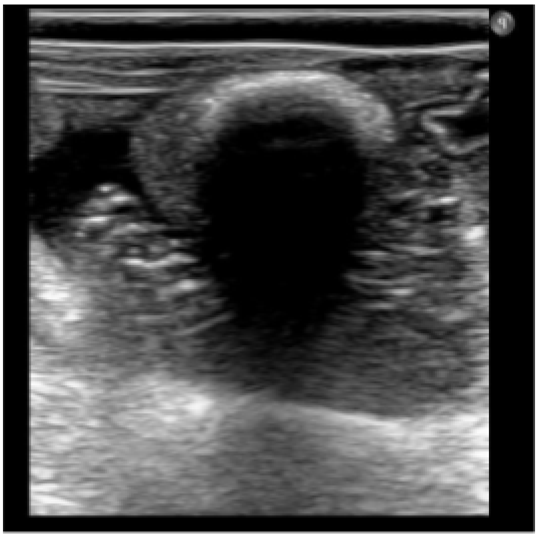 R

Ds

Kidneys

Probe placed dorsally on pelvic limbs, caudally on testicles.

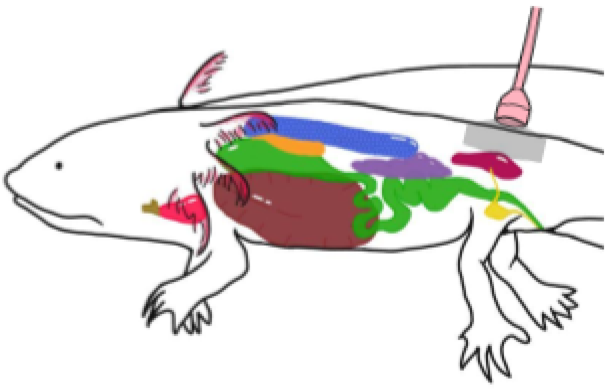

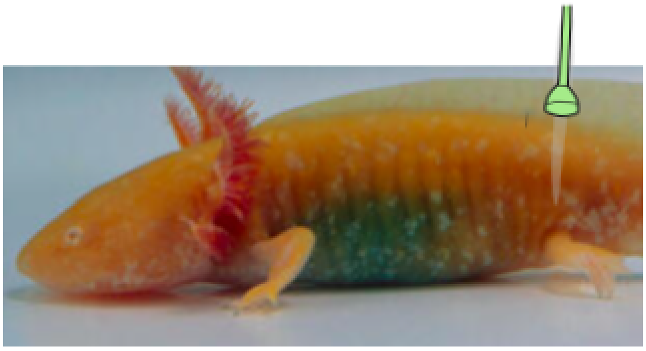

☐ Kidney long axis

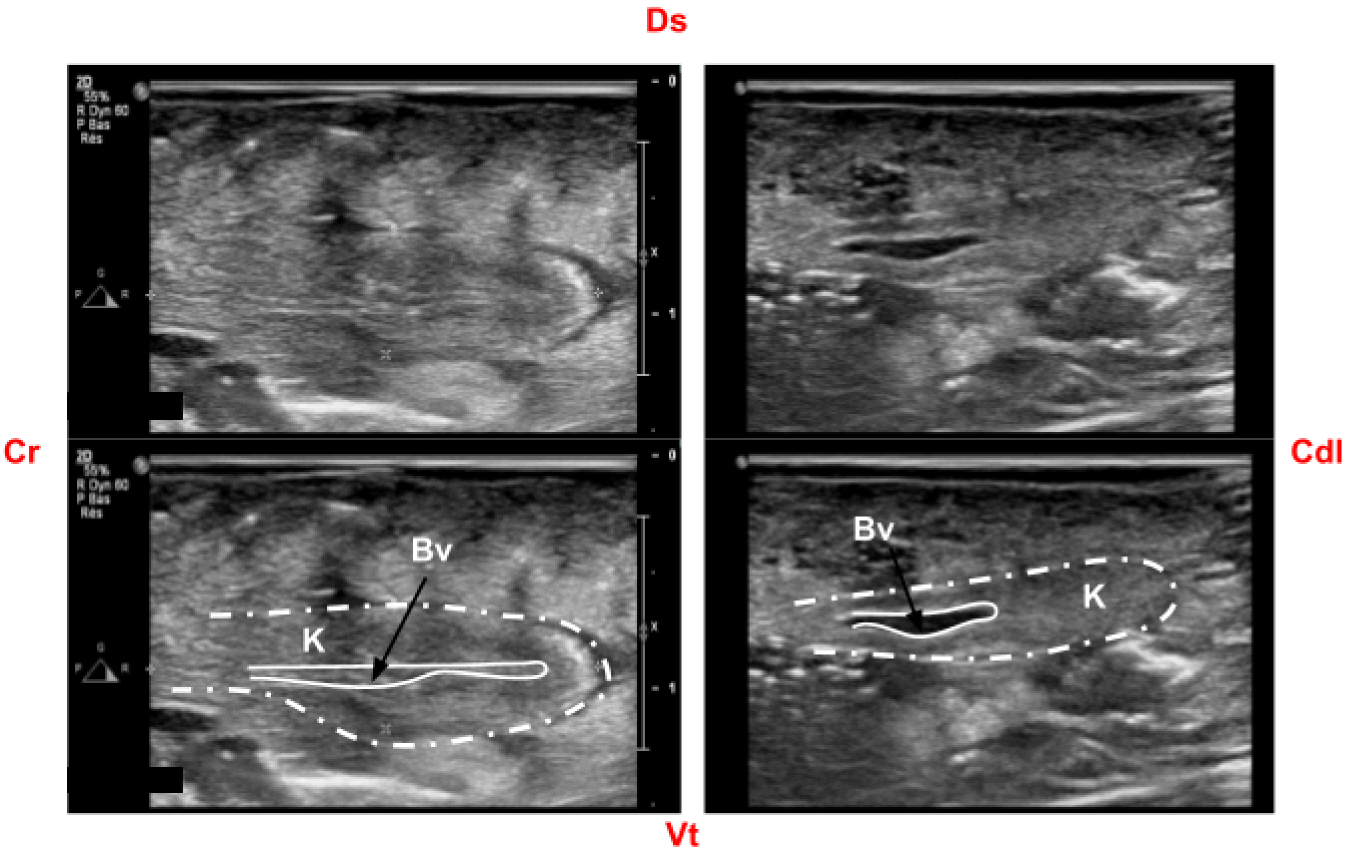

Figure X : Reins, coupe longitudinale
Bv = Blood vessel, **K** = Kidney

☐ Kidney short axis

Probe positioned at 90° to longitudinal kidney section

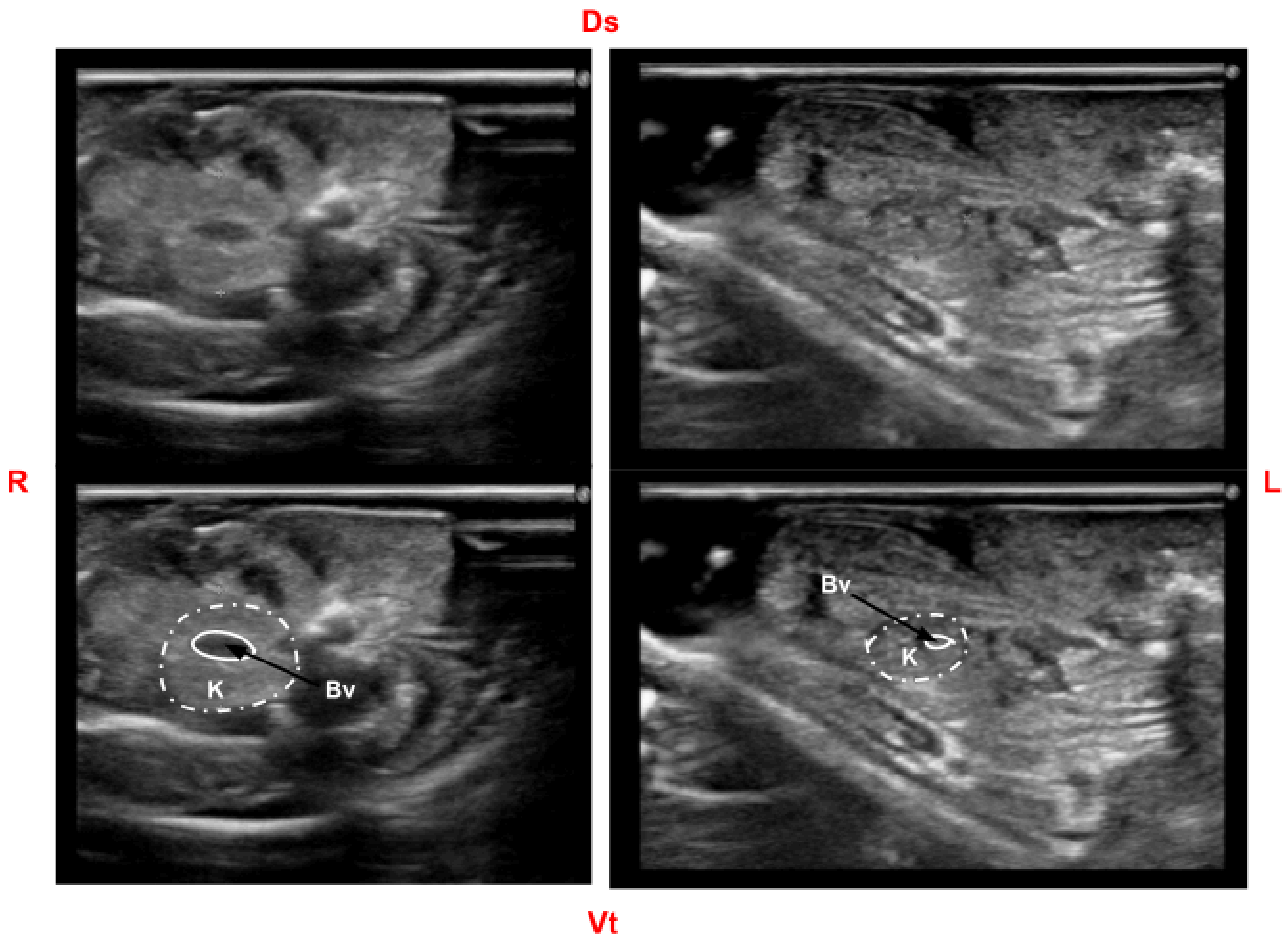

Bv = Blood vessel, **K** = Kidney

Testes

Probe placed dorsally, in the middle of the coelom. The testes are located ventral to the lungs, on either side of the spine.

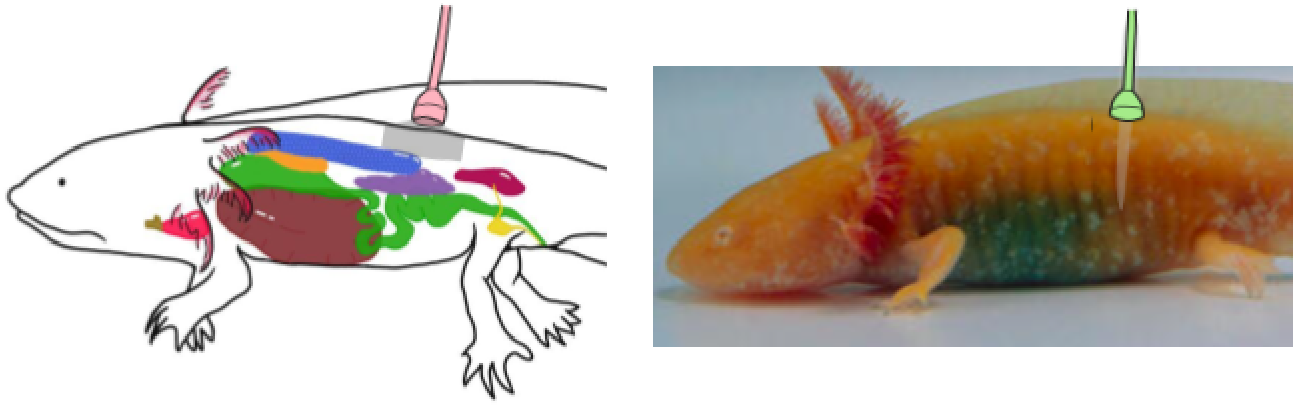

☐ Testicle long axis

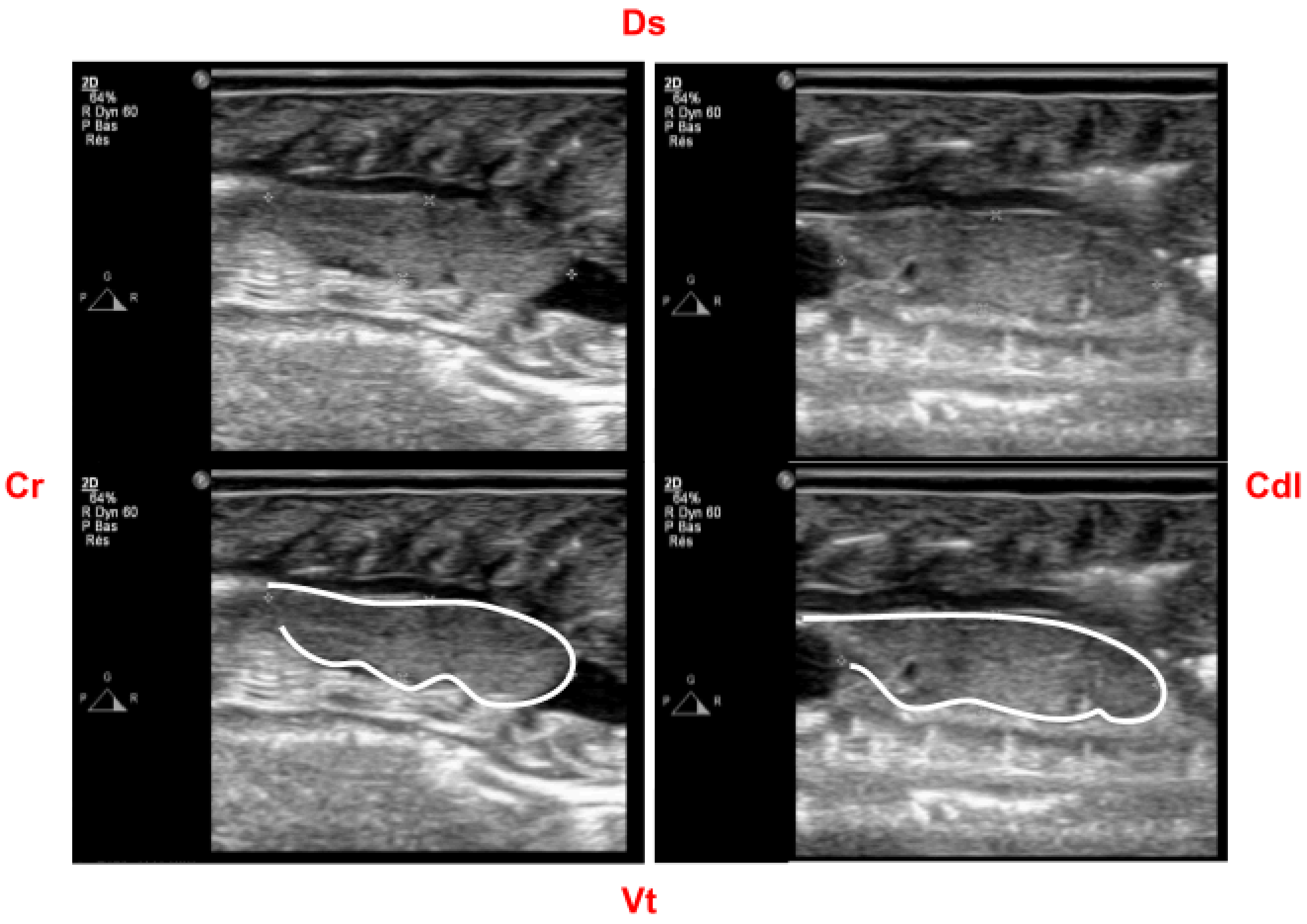

☐ Testicle short axis

Probe positioned at 90° to the longitudinal section of the testicles

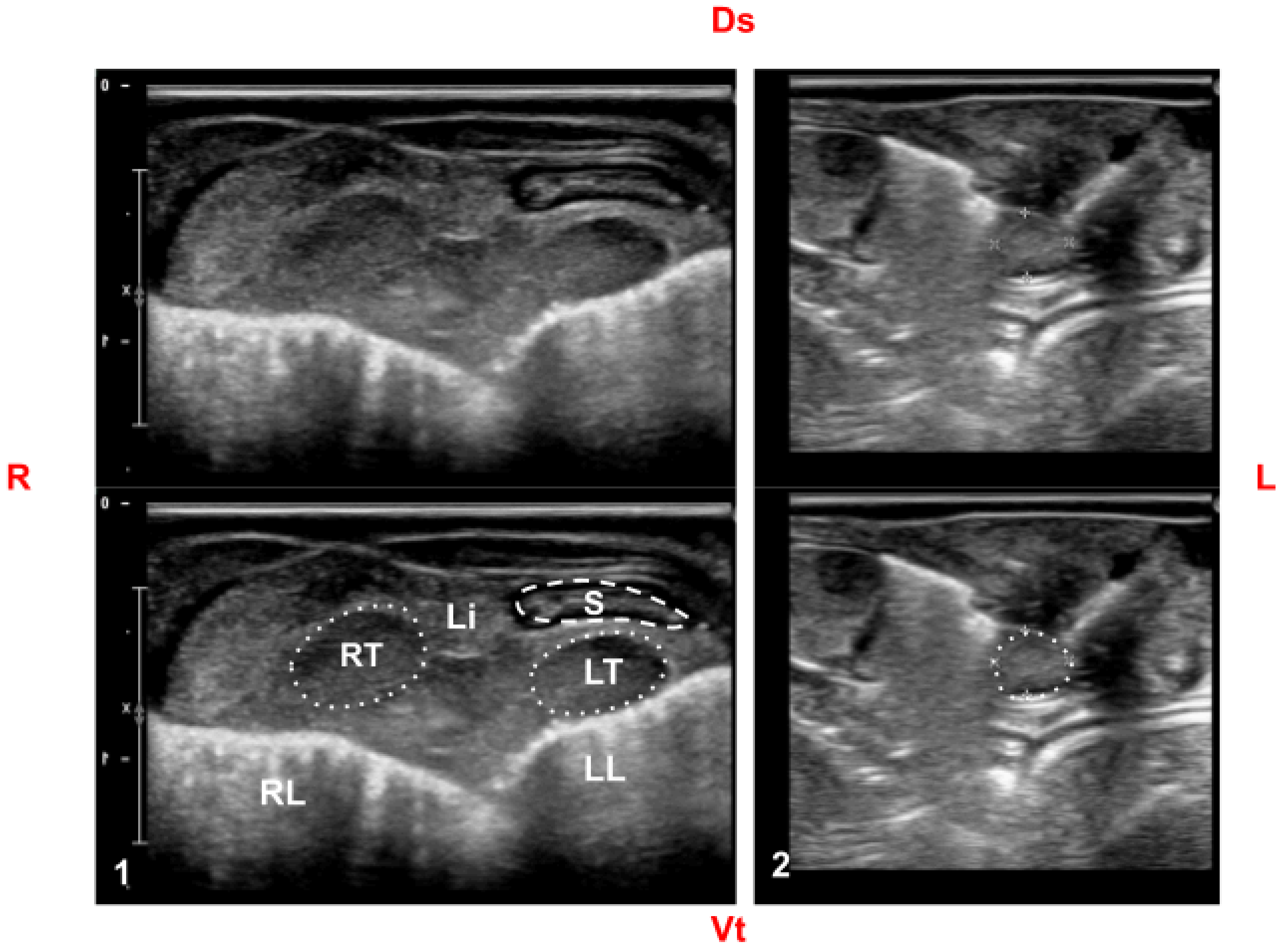

1 : Left and right testes. **2** : Right testes

LT = Left testes, **RT** = Right testes, **LL** = Left lung, **RL** = Right lung, **S** = Stomach, **Li** = Liver

Female genital tract

Probe placed ventrally. Most of the ovaries and oviducts are located caudal to the liver.

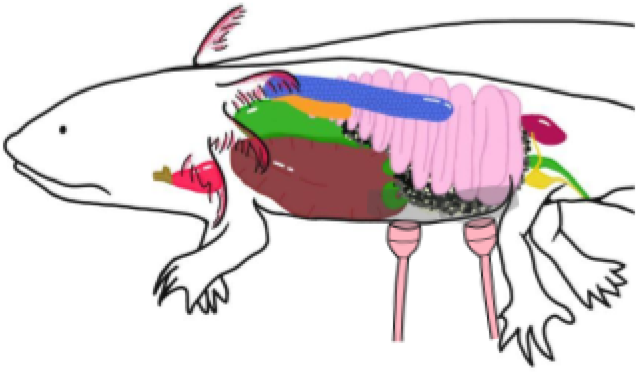

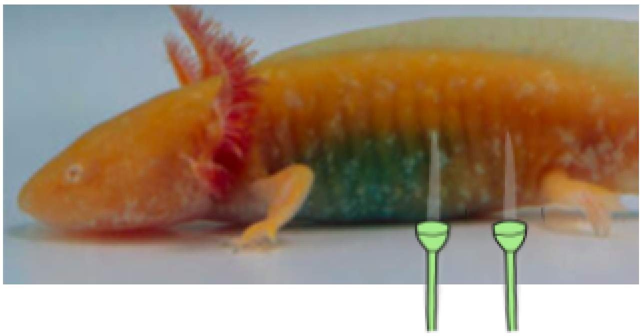

☐ Ovaries and oviducts

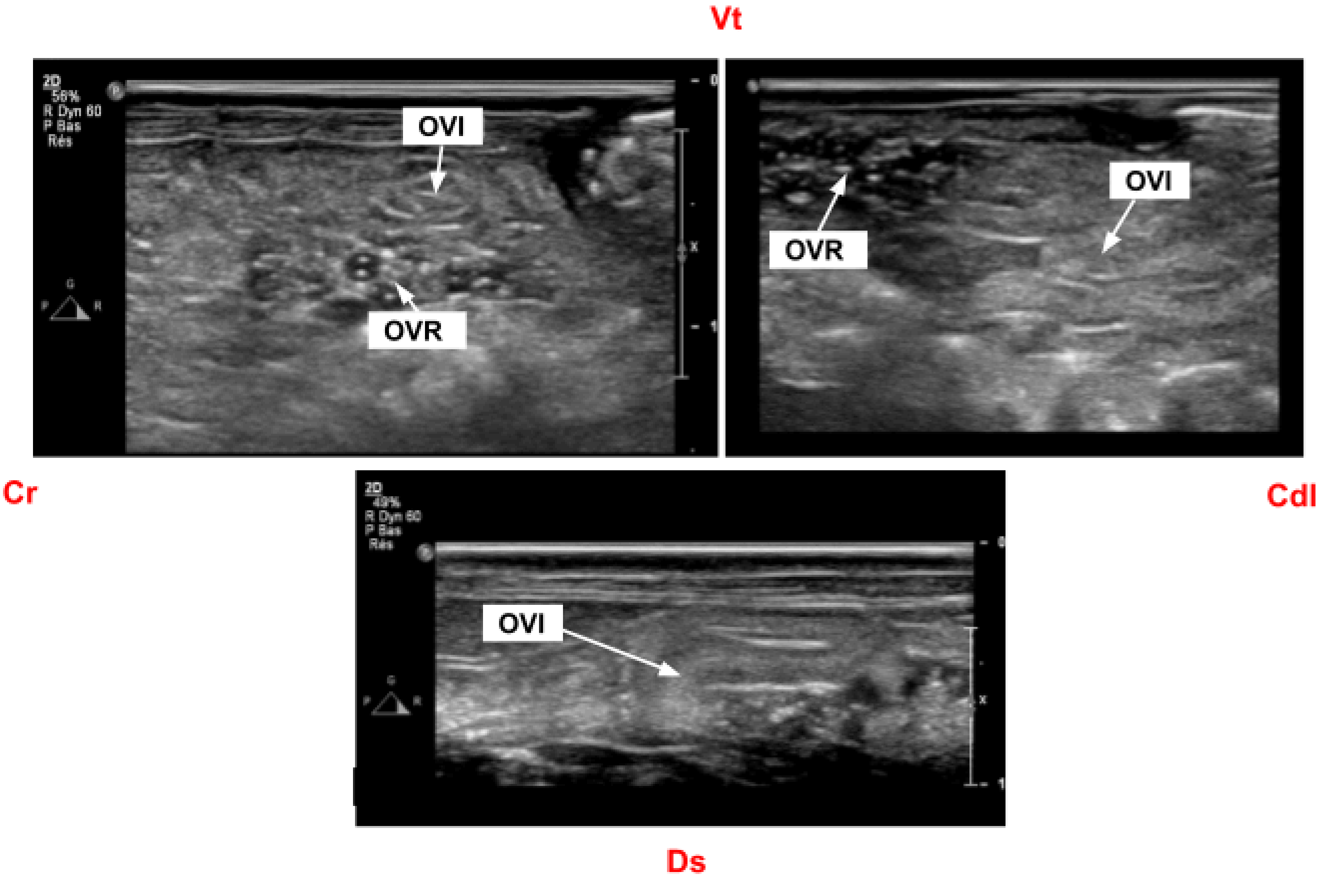

OVI : Oviducts, **OVR** : Ovaries

Lungs

Probe placed dorsally, caudal to the gills. Air induces a reverberation artifact.

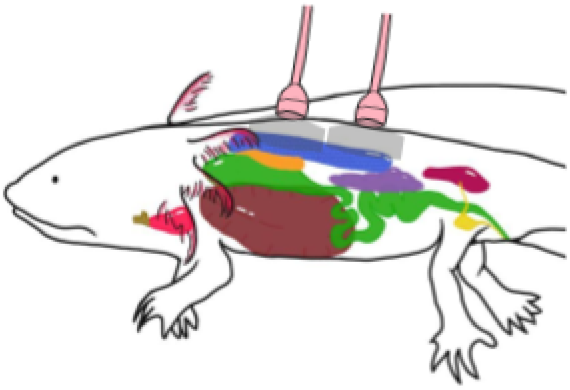

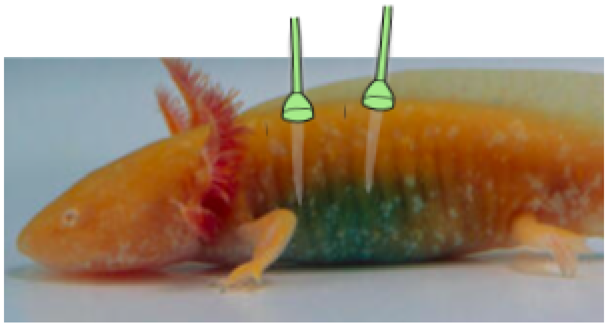

☐ Lung long axis

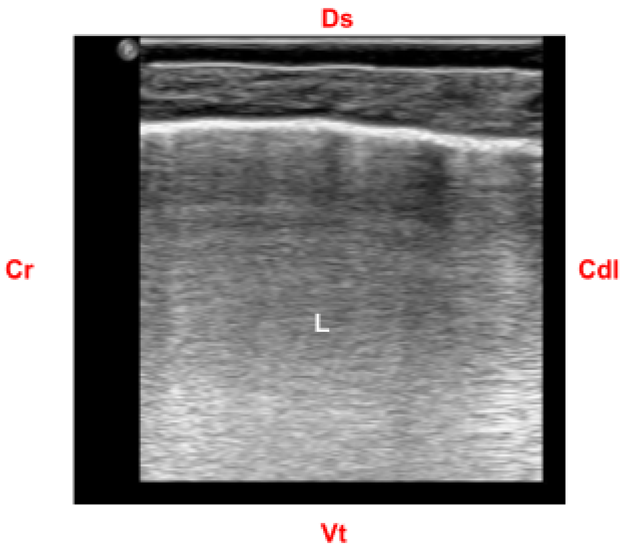

☐ Lung short axis

Probe positioned at 90° to the longitudinal section of the lung

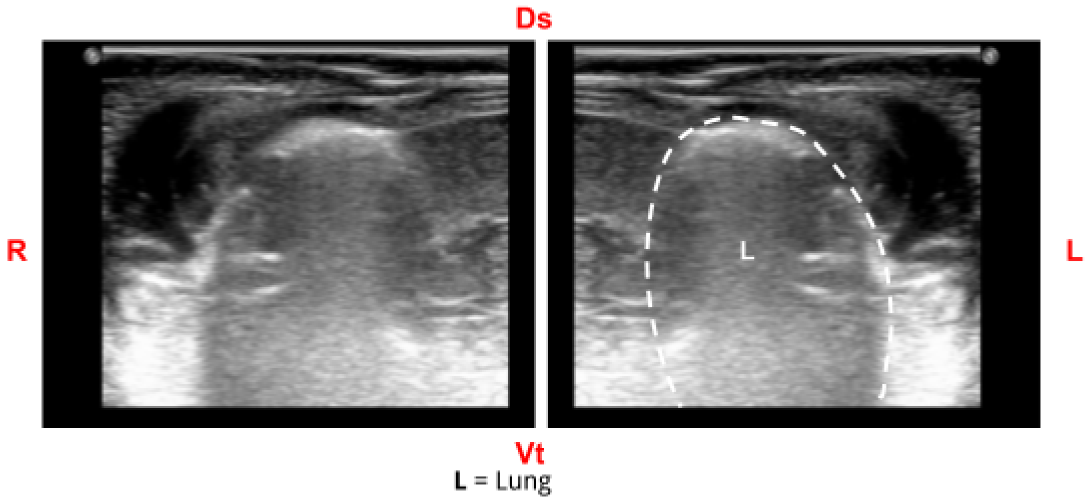

L = Lung

<u>OTHERS STRUCTURES</u>

Rachis

Probe placed dorsally, in median plane

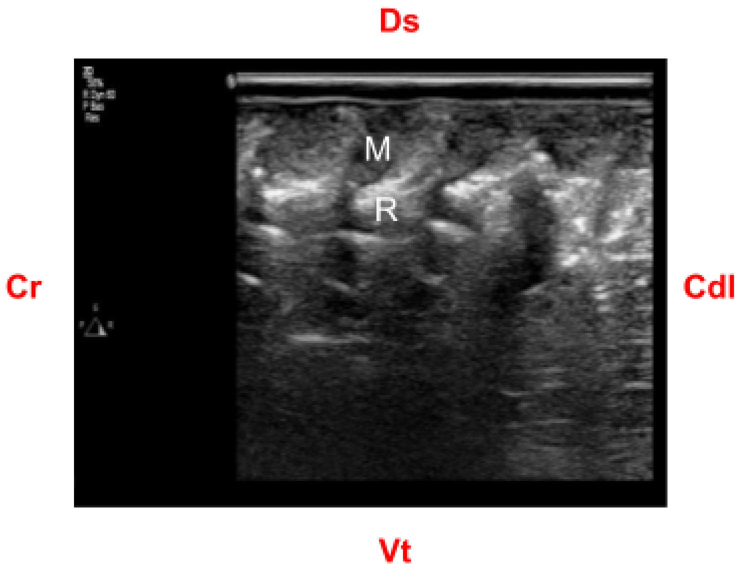

R = Rudimentary ribs **M** = Epaxial muscle

Anechoic fluid

Probe placed ventrally or dorsally, effusion may be localized throughout the coelomic cavity

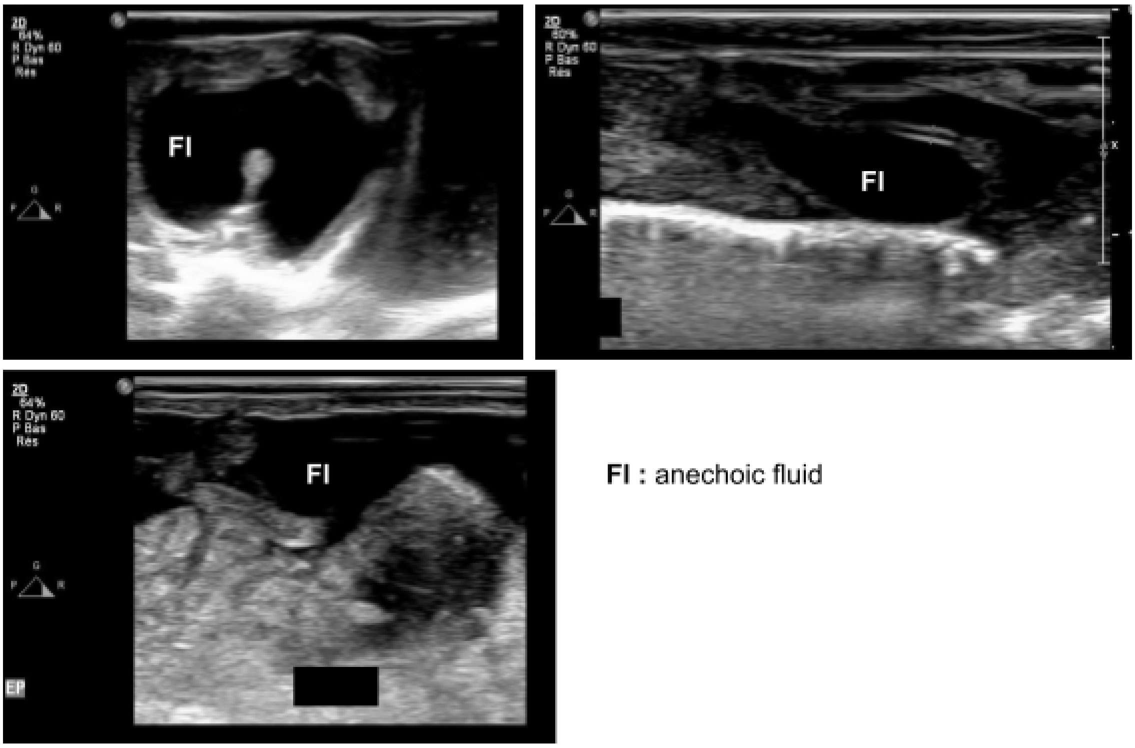

FI : anechoic fluid
